# Distribution of phosphorylated alpha-synuclein in non-diseased brain implicates olfactory bulb mitral cells in synucleinopathy pathogenesis

**DOI:** 10.1038/s41531-023-00491-3

**Published:** 2023-03-25

**Authors:** Bryan A. Killinger, Gabriela Mercado, Solji Choi, Tyler Tittle, Yaping Chu, Patrik Brundin, Jeffrey H. Kordower

**Affiliations:** 1 Graduate College, Rush University Medical Center, Chicago, IL 60612 USA; 2 Parkinson’s disease Center, Department of Neurodegenerative Science, Van Andel Institute, Grand Rapids, MI 49503 USA; 3 ASU-Banner Neurodegenerative Disease Research Center (NDRC), Arizona State University, Tempe, AZ 85287 USA; 4 Pharma Research and Early Development (pRED), F Hoffman-La Roche, New York, NY USA

**Keywords:** Molecular biology, Neurodegeneration, Parkinson's disease, Cell biology

## Abstract

Synucleinopathies are neurodegenerative diseases characterized by pathological inclusions called “Lewy pathology” (LP) that consist of aggregated alpha-synuclein predominantly phosphorylated at serine 129 (PSER129). Despite the importance for understanding disease, little is known about the endogenous function of PSER129 or why it accumulates in the diseased brain. Here we conducted several observational studies using a sensitive tyramide signal amplification (TSA) technique to determine PSER129 distribution and function in the non-diseased mammalian brain. In wild-type non-diseased mice, PSER129 was detected in the olfactory bulb (OB) and several brain regions across the neuroaxis (i.e., OB to brainstem). In contrast, PSER129 immunoreactivity was not observed in any brain region of alpha-synuclein knockout mice. We found evidence of PSER129 positive structures in OB mitral cells of non-diseased mice, rats, non-human primates, and healthy humans. Using TSA multiplex fluorescent labeling, we showed that PSER129 positive punctate structures occur within inactive (i.e., c-fos negative) T-box transcription factor 21 (TBX21) positive mitral cells and PSER129 within these cells was spatially associated with PK-resistant alpha-synuclein. Ubiquitin was found in PSER129 mitral cells but was not closely associated with PSER129. Biotinylation by antibody recognition (BAR) identified 125 PSER129-interacting proteins in the OB of healthy mice, which were significantly enriched for presynaptic vesicle trafficking/recycling, SNARE, fatty acid oxidation, oxidative phosphorylation, and RNA binding. TSA multiplex labeling confirmed the physical association of BAR-identified protein Ywhag with PSER129 in the OB and in other regions across the neuroaxis. We conclude that PSER129 accumulates in the mitral cells of the healthy OB as part of alpha-synuclein normal cellular functions. Incidental LP has been reported in the OB, and therefore we speculate that for synucleinopathies, either the disease processes begin locally in OB mitral cells or a systemic disease process is most apparent in the OB because of the natural tendency to accumulate PSER129.

## Introduction

Synucleinopathies such as Parkinson’s disease (PD), multiple systems atrophy (MSA), and dementia with Lewy bodies (DLB) are a group of neurodegenerative diseases characterized by intracellular alpha-synuclein aggregates called “Lewy pathology” (LP). In synucleinopathies, the distribution of LP has been suggested to adopt one of several characteristic patterns engaging specific brain regions, which has led to the development of pathology staging schemes^1,2^. The brain structure where LP is most consistently found in the olfactory bulb (OB), where LP is often detected in both the diseased and occasionally in the non-diseased brain (i.e., incidental LP)^2–4^. It remains unclear why the OB commonly exhibits LP in the human brain, but one possibility is this structure may be prone to misfolding and accumulation of alpha-synuclein, especially during normal aging^5^. Hyposmia, a common prodromal symptom of PD/DLB^6–9^, supports the hypothesis that the neurodegenerative process begins in the OB^10,11^ years prior to the onset of motor symptoms in PD or cognitive decline in DLB.

LP are complex molecularly heterogeneous intracellular structures containing proteinase K (PK) resistant filaments made of misfolded alpha-synuclein predominantly phosphorylated at serine 129 (PSER129). LP also contains numerous other proteins (i.e., ubiquitin, p62, etc.) along with fragmented organelles and lipids^12,13^ and a diverse array of alpha-synuclein proteoforms^14^. Although alpha-synuclein is predominantly phosphorylated in LP, it has been estimated that less than 4% of the total endogenous alpha-synuclein pool is phosphorylated^15^. The enrichment of PSER129 in LP strongly suggests this post-translation modification (PTM) plays a role in the disease process, but the exact role of PSER129, pathogenic or endogenous, remains unclear. Investigations have primarily focused on whether PSER129 promotes or inhibits alpha-synuclein accumulation, as targeting the responsible kinases and/or phosphatases may be a viable therapeutic strategy. To date, PSER129 remains an agnostic proxy for the synucleinopathy disease process. The development of sensitive and specific antibodies to PSER129 has facilitated the detection of LP. However, the extent to which PSER129 can be present outside pathological LP aggregates is not clear, and it has been difficult to consistently detect low levels of endogenous PSER129^16^. For example, previous attempts to identify endogenous PSER129 by immunohistochemistry (IHC) have been impeded by high-affinity off-target binding of commercially available antibodies that interfere with on-target detection^16^. Determining the role of endogenous PSER129 will help understand the disease process underlying synucleinopathies.

In this paper, we present the results of several observational studies investigating PSER129 in the brain and OB of healthy mammals.

## Results

### PSER129 immunoreactivity in the mouse brain

**Fig. 1:**
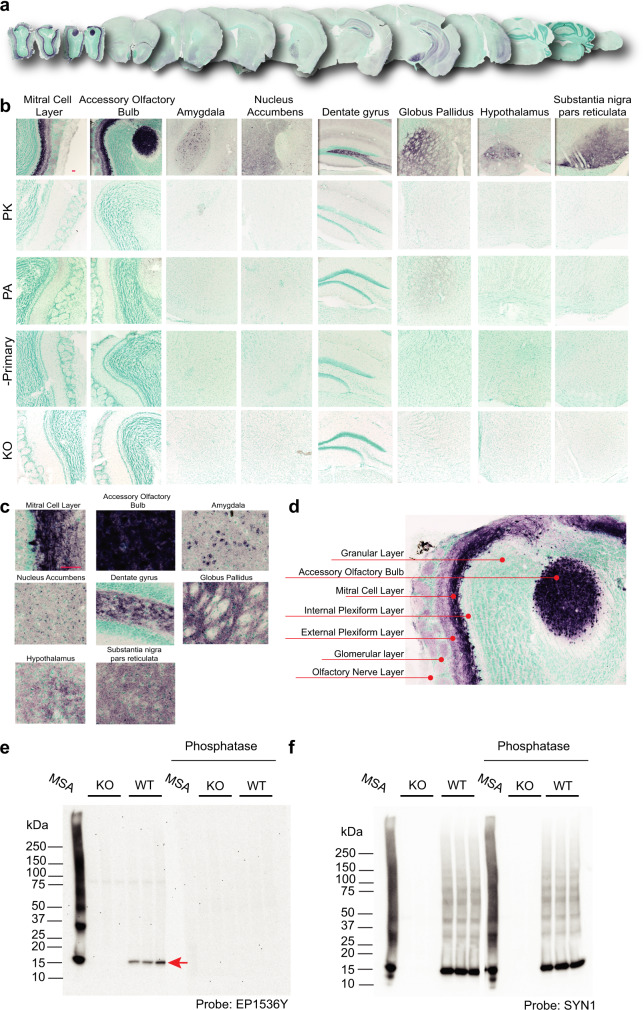
Distribution of PSER129 in the brain of non-diseased mice. **a** Coronal mouse brain sections stained for PSER129 using TSA. **b** Representative images of select brain regions with PSER129 immunoreactivity at high magnification. Tissues digested with PK, incubated with anti-PSER129 antibody preabsorbed against PSER129 (PA), processed without primary antibody (-Primary), and from mice lacking alpha-synuclein (KO). Representative images of select brain regions are shown. Scale bars = 50 microns. WT mice, *n* = 14, KO mice, *n* = 7. **c** Enlarged images from select brain regions showing PSER129 staining patterns in each region. **d** Enlarged image from Fig. b, annotated to show the distribution of PSER129 across layers of the OB. **e** Western blots of proteins extracted from the OB of WT and KO mice probed for PSER129. Blots were conducted in duplicate, with one blot being pretreated with calf intestine alkaline phosphatase (CIAP). **f** Blots reprobed for total alpha-synuclein (antibody “SYN1”). “MSA” denotes a positive control sample. For “MSA”, 20 µg of protein extracted from a single striatal section of an MSA brain was separated and blotted. The red arrow denotes a single 15 kDa PSER129 positive band in WT OB. WT, *n* = 3; KO, *n* = 3.

PSER129 immunoreactivity was regionally specific across the WT mouse brain (Fig. 1a). PSER129 immunoreactivity was most apparent in the OB, but also several other regions showed pronounced PSER129 immunoreactivity, including the accessory OB (AOB), amygdala, nucleus accumbens, dentate gyrus, globus pallidus, hypothalamus, and substantia nigra reticulata (Fig. 1b). The brainstem and cerebellum lacked any discernable immunoreactivity with the notable exception of a group of weakly PSER129 positive cells in the dorsal motor nucleus of the vagus (Supplementary Fig. 1). Apparent nuclear immunoreactivity was observed in many cortical regions and the amygdala. Intense fiber staining was observed in the nucleus accumbens, dentate gyrus, entorhinal cortex, globus pallidus, and substantia nigra pars compacta (Fig. 1b,c). PK digestion prior to IHC abolished PSER129 staining across the neuroaxis (Fig. 1b, “PK”). Immunoreactivity was weak when the primary antibody was preabsorbed against the synthetic PSER129 peptide (Fig. 1b, “PA”), which is consistent with the competitive binding of preabsorbed monoclonal antibodies (Supplementary Fig. 2F, G)^17^. Staining was totally absent when the primary antibody was omitted from the staining protocol (Fig. 1b “-Primary”). PSER129 appeared variable between mice, with some brains showing more prominent staining than others. In total, we observed positive PSER129 immunoreactivity in the OB of 13 out of 14 WT mice tested. In contrast, we did not observe PSER129 in 7 alpha-synuclein knockout mice tested in any brain region under any IHC conditions (Fig. 1b, “KO”). In the OB of WT mice, processes and punctate structures were intensely labeled in the mitral cell layer, while in the external plexiform layer (EPL) and glomeruli (GM) were only weakly labeled (See Fig. 1d for annotated reference). Intense labeling of apparent cells and processes were observed in the AOB. PSER129 labeling was nearly absent in the granular layer (GL) and the olfactory nerve layer (ONL). Occasionally fibers projecting from mitral cells were observed in the inner plexiform layer (IPL).

To further investigate the specificity of PSER129 antibody EP1536Y, we performed western blot (WB) on lysates from the mouse OB (Fig. 1e). Results showed a single 15-kDa band in the OB of WT mice that was absent from alpha-synuclein KO mice (Fig. 1e, red arrow). A positive control sample (i.e., lysate from MSA striatum, Fig. 1e “MSA”, Supplementary Fig. 3) showed a major band at 15-kDa, as well as multiple high molecular weight species. Pretreatment of a duplicate blot with calf intestine alkaline phosphatase (Fig. 1e, “Phosphatase”) abolished EP1536Y reactivity. We then probed for total alpha-synuclein using the “SYN1” antibody (Fig. 1f) and observed a major band at 15-kDa in MSA and OB of WT mice as well as other high molecular weight species. Reactivity was not observed in alpha-synuclein KO mice and “SYN1” reactivity was similar with or without CIAP pretreatment.

### PSER129 occurs as punctate structures in mitral cells

**Fig. 2:**
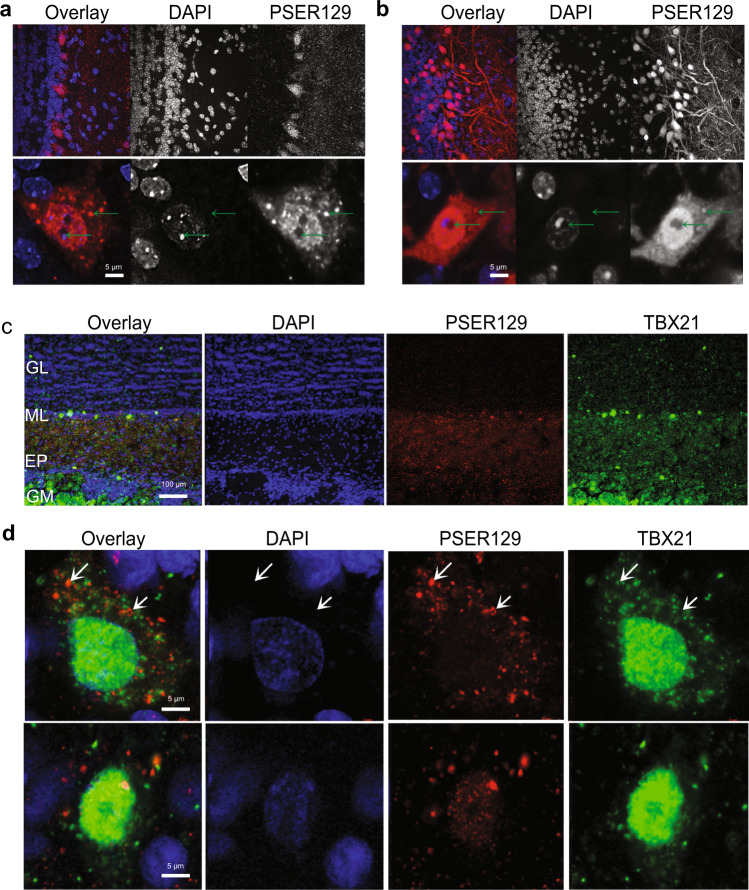
Localization and intracellular distribution of PSER129 in the non-diseased OB. **a** PSER129 was fluorescently labeled using TSA in WT mice. Tissues were counterstained with DAPI. Representative images showing the distribution of PSER129 in the OB. **b** Non-pathology bearing M83 mice labeled for PSER129. Non-human primate OB was dual-labeled for PSER129 and mitral cell marker TBX21. **c** Low magnification confocal images showing regional distribution in non-human primate OB. **d** High-magnification confocal images showing PSER129 puncta in TBX21-positive mitral cell. Green arrows denote intracellular punctate structures, both perinuclear and nuclear. WT mice *n* = 5, M83 mice *n* = 2, non-human primates = 2.

We wanted to determine the intracellular distribution of PSER129 in the OB mitral cell layer. To do this, we fluorescently labeled PSER129 in the OB of WT and M83 mice. Results confirmed the PSER129 signal in the mitral cell layer and diffuse punctate labeling in the outer plexiform layer of WT mice. Punctate labeling was observed throughout the plexiform layer. Cells within the mitral cell layer showed PSER129 granular staining in the nucleus and perinuclear compartments. (Fig. 2a, b, green arrows). PSER129 and DAPI weakly co-localized in these cells. M83 mice showed more prominent PSER129 labeling of the mitral cell layer and strong fibers labeling in the EPL (Fig. 2b). Within the mitral layer cells, strong nuclear and perinuclear PSER129 labeling. Punctate PSER129 labeling in the EPL extended to the glomerulus. PSER129 punctate staining was sporadically observed in the GL of WT mice.

**Fig. 3:**
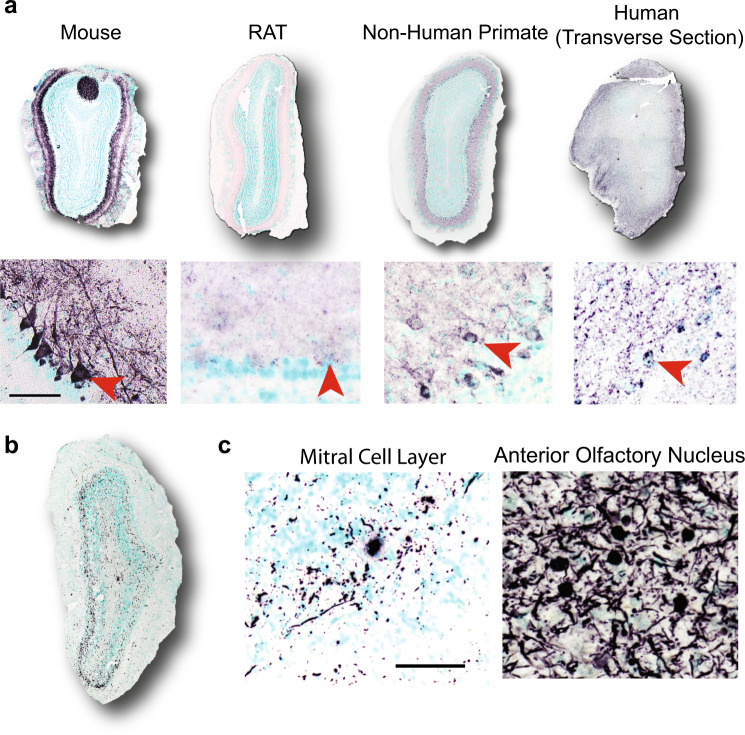
Mitral cell PSER129 is conserved across species. **a** Representative images of PSER129 staining in OB sections from mice, rats, non-human primates, and humans. All sections were coronal, except for human OB, which was a section in the transverse plane. Depicted below each OB are high-magnification images of staining in the mitral layer. Scale bar = 50 µ. Red arrowheads indicate positive PSER129 mitral cells. **b** Representative images of PSER129 staining in PD/DLB OB. Section from the main bulb depicted. **c** High-magnification images show PSER129 staining in the mitral cell layer and anterior olfactory nucleus. Scale bar = 50 µ.

To determine if the punctate PSER129 staining originated in mitral cells, we fluorescently labeled both PSER129 and the mitral cell marker T-box transcription factor 21 (TBX21) using the TSA multiplex protocol. This experiment was performed in the OB from healthy non-human primates because the anti-TBX21 antibody used is of mouse origin, and therefore performing this experiment on mouse tissues potentially would produce ambiguous results. We had already observed the same PSER129 staining pattern in the non-human primate OB (see Fig. 3a and Supplementary Fig. 4) and therefore chose to assess PSER129 colocalization in this tissue. Results showed that TBX21 labeling was predominantly nuclear in select cells of the mitral layer (Fig. 2c, d). TBX21 labeling was observed in some perinuclear structures. Perinuclear PSER129 puncta were observed in TBX21-positive mitral cells. Within mitral cells, PSER129 and TBX21 did not colocalize (Fig. 2d).

### Mitral cell PSER129 is conserved across mammalian species

Next, we wanted to determine if PSER129-positive mitral cells were conserved across species. To do this, we stained for PSER129 in the OB of healthy rats, non-human primates, healthy adults, and individuals diagnosed with PD (see Table 1 for summary, for detailed human data, see Supplementary Data 6). Results showed mitral cell staining in rats, non-human primates, and humans, like that which was observed in mice (Fig. 3a). Antibody clone psyn#64 also detected PSER129 in mitral cells similar to what we observed with antibody EP1536Y (Supplementary Fig. 4). In the OB of human PD, we found that HIAR (Heat-induced antigen retrieval) or PK digestion enhanced PSER129 detection, and in the healthy OB HIAR often enhanced PSER129 staining (Supplementary Fig. 5). PSER129 staining of the PD OB shows mostly processes baring pathology, with a few cell bodies being immunoreactive. PSER129 positive inclusions could be detected across all layers of the OB, including the mitral cell layer, and most prominently in the anterior olfactory nucleus (Fig. 3b, c). We attempted to verify that OB mitral cells were intact in all samples by staining with the TBX21 antibody (Supplementary Fig. 6); however, we found immunoreactivity was absent from several human samples (Table 1), which has been reported previously and may result from differences in postmortem interval^18^.

**Table 1:** List of specimens and summary of results.

#	Species	Sex	Age at death (yrs)	Method of fixation	Strain	Genotype	EP1536Y staining*	TBX21 staining†	Disease
1	*Mus musculus*	M	0.66	PF/DF	C57BL/6 J	α-syn KO	0	1	NA
2	*Mus musculus*	M	0.66	PF/DF	C57BL/6 J	α-syn KO	0	1	NA
3	*Mus musculus*	M	0.66	PF/DF	C57BL/6 J	α-syn KO	0	1	NA
4	*Mus musculus*	M	0.66	PF/DF	C57BL/6 J	α-syn KO	0	1	NA
5	*Mus musculus*	M	0.66	PF/DF	C57BL/6 J	WT	1	1	NA
6	*Mus musculus*	M	0.66	PF/DF	C57BL/6 J	WT	1	1	NA
7	*Mus musculus*	M	0.66	PF/DF	C57BL/6 J	WT	0	1	NA
8	*Mus musculus*	M	0.66	PF/DF	C57BL/6 J	WT	1	1	NA
9	*Mus musculus*	M	0.83	PF/DF	C57BL/6 J	WT	1	ND	NA
10	*Mus musculus*	M	0.83	PF/DF	C57BL/6 J	WT	1	ND	NA
11	*Mus musculus*	M	0.83	PF/DF	C57BL/6 J	WT	1	ND	NA
12	*Mus musculus*	F	0.66	PF/DF	C57BL/6 J	α-syn KO	0	1	NA
13	*Mus musculus*	F	0.66	PF/DF	C57BL/6 J	α-syn KO	0	1	NA
14	*Mus musculus*	F	0.66	PF/DF	C57BL/6 J	α-syn KO	0	1	NA
15	*Mus musculus*	F	0.66	PF/DF	C57BL/6 J	WT	1	1	NA
16	*Mus musculus*	F	0.66	PF/DF	C57BL/6 J	WT	1	1	NA
17	*Mus musculus*	F	0.66	PF/DF	C57BL/6 J	WT	1	1	NA
18	*Mus musculus*	F	0.66	PF/DF	C57BL/6 J	WT	1	1	NA
19	*Mus musculus*	F	0.66	PF/DF	C57BL/6 J	WT	1	1	NA
20	*Mus musculus*	F	0.83	PF/DF	C57BL/6 J	WT	1	ND	NA
21	*Mus musculus*	F	0.83	PF/DF	C57BL/6 J	WT	1	ND	NA
22	*Rattus norvegicus domestica*	NK	1.33	DF	SD	WT	0	1	NA
23	*Rattus norvegicus domestica*	NK	1.33	DF	SD	WT	1	1	NA
24	*Rattus norvegicus domestica*	NK	1.33	DF	SD	WT	1	1	NA
‡25	*Macaca fascicularis*	M	10.86	PF/DF	NA	WT	1	1	NA
26	*Macaca fascicularis*	F	5.59	PF/DF	NA	WT	1	1	NA
27	*Homo sapiens*	F	93.24	DF	NA	NA	0	0	NS
28	*Homo sapiens*	M	87.8	DF	NA	NA	0	ND	NS
29	*Homo sapiens*	F	74.1	DF	NA	NA	0	0	NS
30	*Homo sapiens*	F	87	DF-Paraffin	NA	NA	1	1	NS
31	*Homo sapiens*	M	69	DF	NA	NA	0	0	PD/DLB
32	*Homo sapiens*	F	70	DF	NA	NA	0	0	PD/DLB
33	*Homo sapiens*	M	86	DF	NA	NA	0	0	PD/DLB
34	*Homo sapiens*	M	81	DF	NA	NA	0	0	PD/DLB
35	*Homo sapiens*	M	NK	DF	NA	NA	0	0	PD/DLB
§36	*Homo sapiens*	M	NK	DF	NA	NA	1	1	PD/DLB

*NA* not assessed, *NK* not known, *NS* non-synucleinopathy, *ND* not determined, *PF/DF* perfusion fixation/drop fixation.

*Antibody EP1536Y was used to detect PSER129 in the OB. 0 means PSER129 positive cells were not observed in the mitral cell layer and 1 denotes PSER129 positive cells were observed in the mitral cell layer. Only staining conditions without proteinase K were considered.

^†^TBX21 is a mitral cell marker. 0 means no staining and 1 means staining.

^‡^Specimen 25 was euthanized early due to post-op-right subdural hematoma.

^§^Specimen 36 had weak TBX21 staining but was observed in the citrate retrieval condition.

### PSER129 associates with PK-resistant alpha-synuclein in mouse mitral cells

**Fig. 4:**
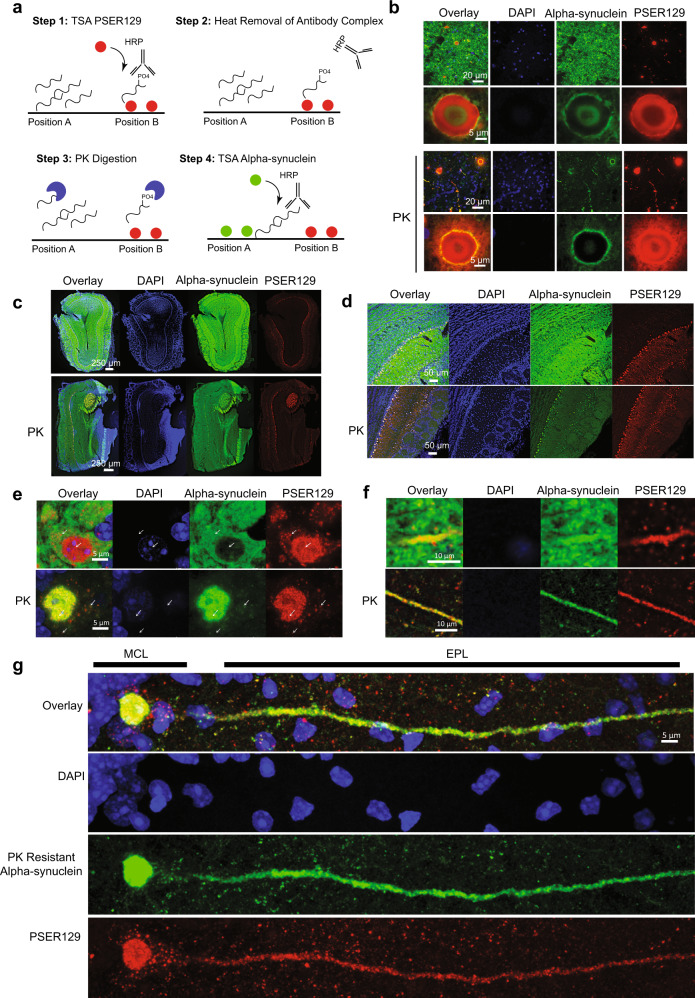
PSER129 associates with PK-resistant alpha-synuclein in the mouse OB. **a** Summary of approach for measuring PK resistance in situ. Step 1, the location of PSER129 is labeled using TSA. CF-568 dye is covalently bound to tissue (Position “B”). Step 2, low pH and heat are used to remove the antibody complex from the tissue while leaving the CF-568 dye intact. Step 3, tissues are briefly treated with PK to destroy enzyme-accessible epitomes of alpha-synuclein. Step 4, an antibody against total alpha-synuclein is used for TSA labeling of the remaining alpha-synuclein in the sample. (Position “A”). Results allow simultaneous visualization of PK-sensitive and PK-resistant epitopes. **b** PD brain sections processed with this dual-labeling protocol. High-magnification confocal images of the substantia nigra. The top panels depict results without PK and below with PK digestion (“PK”). **c** Low magnification confocal images of OB dual-labeled for PSER129 and alpha-synuclein. The top panels show an OB section processed in the absence of PK. The bottom panels depict OB sections processed with PK. **d** High-magnification images of the mitral cell layer. Expanded high-magnification images depicting a mitral cell body (**e**) and apical dendrite (**f**) in the EPL with (bottom panel) and without PK (top panel). **g** Distribution of PSER129 and PK-resistant alpha-synuclein in a single OB mitral cell. MCL mitral cell layer, EPL external plexiform layer. (WT mice *n* = 3, PD substantia nigra = 3).

In human synucleinopathies and in synucleinopathy models, aggregated alpha-synuclein is predominantly phosphorylated^19^. Previously we observed that PSER129 in the mouse OB was PK-sensitive (Fig. 1b, “PK”), suggesting that this alpha-synuclein pool was not aggregated. However, we previously observed unusual differential labeling in OB mitral cells, where “SYN1” antibody reactivity in mitral cells appeared to be inversely proportional to PSER129 immunoreactivity, suggesting that “SYN1” alpha-synuclein epitope (a.a. 91–98) was inaccessible in PSER129 positive accumulations (Supplementary Fig. 7). This suggested that alpha-synuclein associated with PSER129 or PSER129 itself might be in complex or aggregated. To experimentally test this possibility, we developed and used a new TSA multiplexing approach to label PK-resistant alpha-synuclein and PSER129 in the same tissue, while avoiding destroying the PSER129 epitope (Fig. 4a). This technique allowed us to assess the distribution of PK-sensitive PSER129 in relation to PK-resistant alpha-synuclein.

We validated this approach by applying it to PD substantia nigra tissues (Fig. 4b). Without PK digestion, alpha-synuclein signal is abundant and overlaps with PSER129 primarily in Lewy bodies and Lewy neurites (Fig. 4b, top panels). Samples processed with PK digestion show markedly reduced alpha-synuclein signal, with the remaining alpha-synuclein overlapping with PSER129. Interestingly, the complete overlap between alpha-synuclein and PSER129 was not observed, even after PK digestion, suggesting these two antibodies preferentially label different alpha-synuclein pools, a phenomenon that has been described in several other reports^14,20^ and observed here (Supplementary Fig. 7).

We then applied this technique to mouse OB to determine if PSER129 was associated with PK-resistant alpha-synuclein. Results showed high alpha-synuclein content throughout the layers of the OB (Fig. 4c, d, top panels) when PK was omitted from the protocol. Alpha-synuclein immunoreactivity was decreased in the OB following PK digestion, with regional immunoreactivity being preserved particularly in the mitral cell layer, AOB, and apparent fibers of the OB tract (Fig. 4c, d, bottom panels “PK”). High overlap between PSER129 and PK-resistant alpha-synuclein was observed, particularly in the mitral cell layer and AOB, but to a lesser extent in the fibers of the OB tract, which were poorly labeled with PSER129 but clearly labeled with PK-resistant alpha-synuclein. High-magnification images of the mitral cells and mitral cell projections (Fig. 4e, f) showed selective overlap between the two signals. Without PK, PSER129 overlapped well with total alpha-synuclein in projections of the EPL (Fig. 4f, top panel), but in the mitral cell body overlap was minimal with cytosolic PSER129 puncta and nuclear PSER129 being spatially segregated from total alpha-synuclein (Fig. 4e, top panel, white arrows). Remarkably, with PK digestion, total alpha-synuclein and PSER129 then showed high overlap in mitral cells, particularly in the nucleus and projections (Fig. 4e, f, Bottom panels, “PK”). Cytosolic PSER129 puncta were associated with faint PK- resistant alpha-synuclein immunoreactivity (Fig. 4e, “PK”, white arrows and Fig. 4g). Projection images of single mitral cells showed small perinuclear PSER129 puncta and PSER129 surrounding mitral cells that were not associated with PK-resistant alpha-synuclein. Within the dendrite and nucleus, PSER129 and PK-resistant alpha-synuclein were closely, but not completely, associated.

### PSER129 in mitral cells was not associated with ubiquitin

**Fig. 5:**
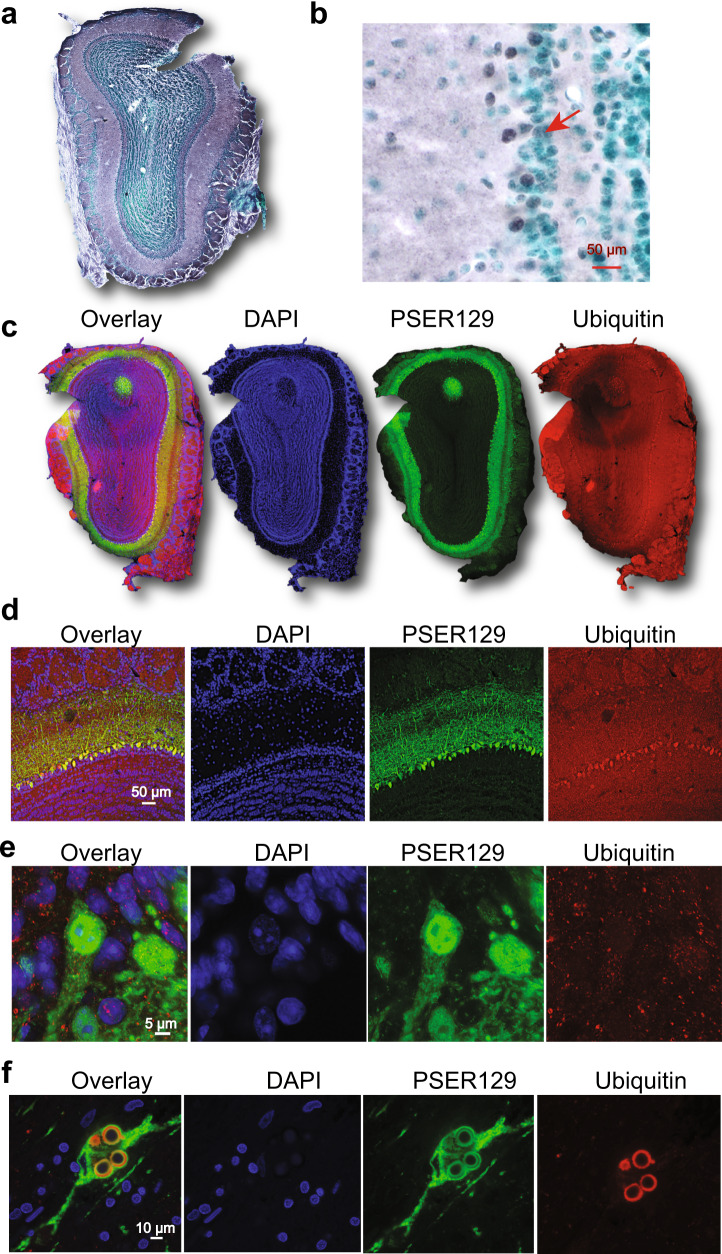
PSER129 is not associated with ubiquitin in OB mitral cells. Ubiquitin levels and distribution in the mouse OB were determined. **a** Low magnification image showing ubiquitin staining in the main OB. **b** High-magnification image showing ubiquitin staining in the mitral cell layer. The red arrow denotes ubiquitin-positive cells in the mitral cell layer. **c** Low magnification images of OB sections fluorescently labeled for PSER129 and ubiquitin. **d** High-resolution confocal images showing the distribution of ubiquitin in the mitral cell layer. **e** Confocal image of single PSER129 positive mitral cell. **f** Substantia nigra from PD brain fluorescently labeled for ubiquitin and PSER129 using the same TSA multiplex approach. All sections were counterstained with either methyl green or DAPI (WT mice *n* = 3, PD substantia nigra *n* = 3).

Intraneuronal ubiquitin can accumulate with age and disease^21^ and is enriched in LP, particularly mature Lewy bodies^22^. To determine if PSER129 positive mitral cells also accumulated ubiquitin, we multiplex labeled ubiquitin and PSER129 in the mouse OB. Results showed that ubiquitin staining was observed throughout the layers of the OB (Fig. 5a). Cells near or within the mitral layer were labeled for ubiquitin (Fig. 5b). Overlap between PSER129 and ubiquitin was observed with confocal images taken at lower magnifications (Fig. 5c, d). However, high-magnification confocal images showed little overlap between PSER129 and ubiquitin in mitral cells (Fig. 5e). Ubiquitin could be found in both the cytosol and nucleus of PSER129 mitral cells. Using the same multiplexing protocol, we found that, as expected, ubiquitin and PSER129 intensely labeled mature Lewy bodies in the PD substantia nigra (Fig. 5f). PSER129 also labeled structures peripheral to Lewy bodies (Fig. 5f). Together, these results demonstrated that unlike mature LP, in mitral cells PSER129 was not strongly associated with ubiquitin accumulation.

### PSER129 interacts with vesicle cycle machinery in the OB

**Fig. 6:**
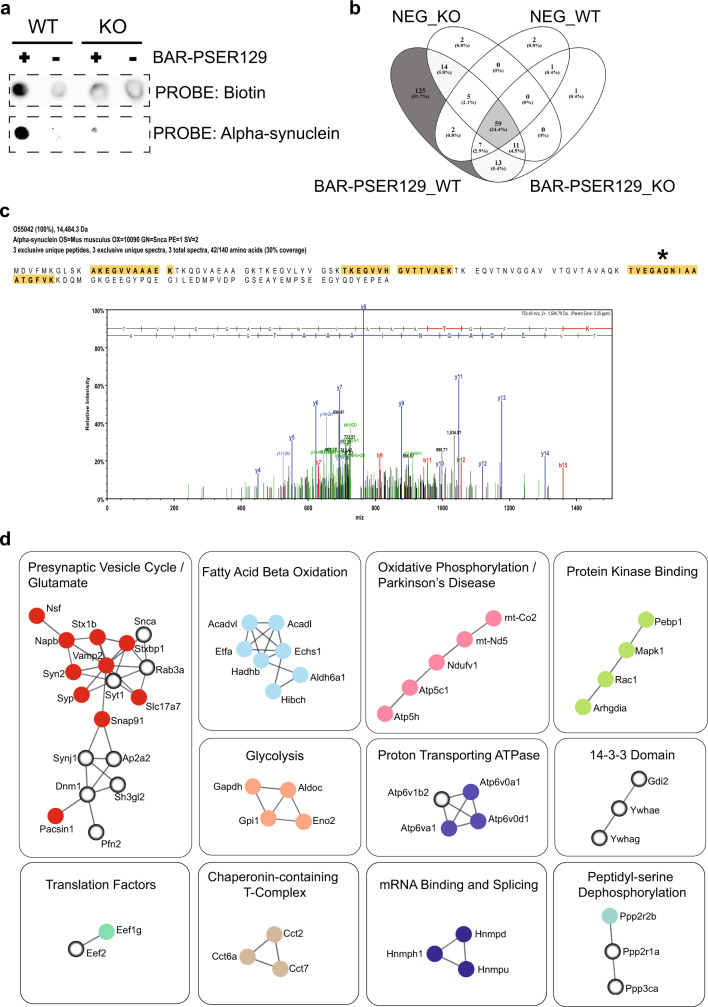
PSER129 interactome in mouse OB. BAR-PSER129 was conducted on brain sections from WT and KO mice. A primary antibody omission control was conducted for each sample (“Neg”), where the samples were processed in the absence of the PSER129 antibody. **a** Spot blot of the captured proteins were probed for biotinylated proteins or alpha-synuclein. The resulting chemiluminescent detection is shown. **b** Samples were analyzed by LC-MS/MS. Venn diagram depicts the overlap of identified proteins between samples. 125 proteins were identified exclusively in the BAR-PSER129 WT sample. **c** Alpha-synuclein identification in the BAR-PSER129 capture sample. Three high-confidence peptides (highlighted yellow) were identified. Spectrum from the most abundant alpha-synuclein peptide identified. (Denoted by an asterisk). **d** STRING analysis and MCL clustering of the 125 proteins identified. High-confidence functional interactions are shown (>0.7). Proteins lacking high-confidence functional interactions are not shown. Enrichment analysis was performed for each distinct STRING cluster and a consensus-enriched term(s) were manually annotated above each cluster. Protein nodes are colored for easy visualization of each cluster. Open-circle nodes indicate alpha-synuclein interactors previously identified in rat primary cortical neurons using in vivo proximity labeling methods^24^.

We had observed PSER129 staining in healthy OB, and this observation provided a unique opportunity to study endogenous PSER129. To determine the functional relevance of the apparent endogenous PSER129 pool, we measured the PSER129 interactome in the OB using a proximity labeling technique we previously used to identify pathological alpha-synuclein interactions in fixed tissues called BAR-PSER129^23^. We applied BAR-PSER129 to both WT and alpha-synuclein KO mice. Dot blot following BAR-PSER129 showed enrichment for biotinylated proteins and alpha-synuclein (Fig. 6a) only in WT mice. BAR-PSER129 conducted in the absence of a primary antibody (“Neg” or “−”) showed minimal signal in WT and KO mice.

Next, we performed liquid chromatography-tandem mass spectrometry (LC-MS/MS) of the BAR-PSER129 samples and identified a total of 241 proteins (Supplemental Data 1), with 125 proteins (51.7% of those identified) being unique to BAR-PSER129 in WT OB (Fig. 6b and Supplemental Data 3). In agreement with dot blot results (Fig. 6a), alpha-synuclein was exclusively identified in the BAR-PSER129 WT OB condition, with three exclusive unique spectra comprising a 30% coverage of alpha-synuclein protein being detected in this sample (Fig. 6c and Supplemental Data 2). Unbiased hierarchal clustering of identified proteins across samples verified BAR-PSER129 WT OB sample segregated from all other capture conditions (Supplemental Fig. 8). Amongst the BAR-PSER129 WT OB, unique proteins were four kinases (Camk2b, Pdxk, Mapk1, and Dclk1) and three phosphatases (Ppp3Ca, Ppp2r1a, and Ppp2r2b).

To infer functional interactions of the 125 BAR-PSER129 identified proteins, we performed a Search Tool for the Retrieval of Interacting Genes/Proteins (STRING) analysis with Markov Clustering (MCL). Results showed 11 clusters of high-confidence (>0.7) functional interactions (Fig. 6d). The function of each interaction cluster was determined and annotated using enrichment analysis. The largest functional cluster consisting of 18 proteins, including the target protein alpha-synuclein, was enriched for “Presynaptic Vesicle Cycle / Glutamate”. Other clusters included “fatty acid beta-oxidation”, “oxidative phosphorylation/Parkinson’s disease”, “protein kinase binding”, “glycolysis”, “proton transporting ATPase”, “14-3-3 domain”, “peptidyl-serine dephosphorylation”, “chaperonin-containing T-complex”, “mRNA binding and splicing”, and “translation factors.” Enrichment analysis of the 125 identified proteins confirmed an overall strong enrichment for presynaptic vesicle processes (false discovery rate (FDR) <1 × 10^−10^ Supplemental Data 4, 5) and 12 of the proteins identified were from the Kyoto Encyclopedia of Genes and Genomes (KEGG) Parkinson’s disease pathway. Several BAR-PSER129 identified proteins (24/125) and functional clusters (5/11) identified here contained previously identified alpha-synuclein interactors^24^ (Fig. 6d, open-circle nodes), while the remaining were distinct.

### Mitral cell PSER129 localizes with Ywhag

**Fig. 7:**
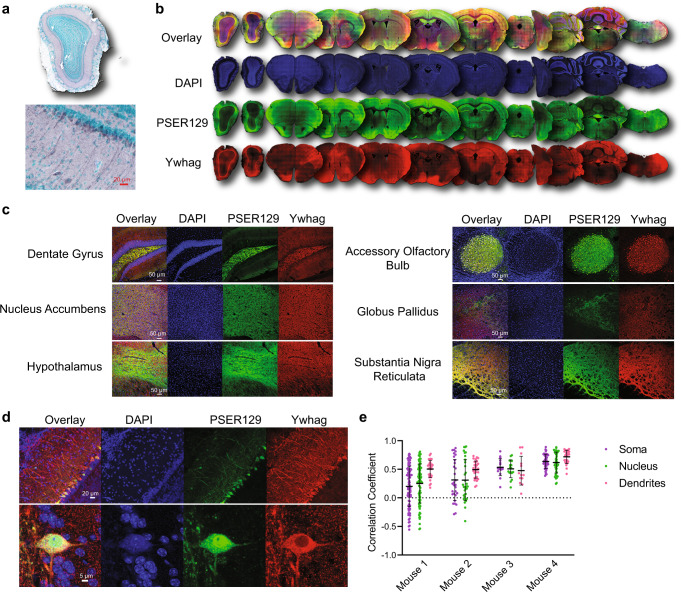
BAR-PSER129 identified protein Ywhag associated with PSER129 across the neuroaxis. **a** IHC detection of Ywhag in the WT mouse OB. **b** Whole section scans of dual-labeled Ywhag and PSER129 across the neuroaxis of a healthy mouse. **c** High-magnification confocal images of Ywhag and PSER129 in select brain regions. **d** High-magnification confocal images of Ywhag and PSER129 in the mitral cell layer. **e** Colocalization analysis of mitral cells. Pearson’s correlation coefficient was calculated in the soma, nucleus, and dendrites of PSER129 positive mitral cells. (mean ± SD, WT mice, *n* = 4).

BAR-PSER129 identified several 14-3-3 proteins, one of which (Ywhag) had been previously found in close association with LP and interacts with alpha-synuclein^25,26^. Furthermore, Ywhag binds phosphoserine motifs raising the possibility that Ywhag directly interacts with PSER129. To verify the BAR-identified interaction between Ywhag and PSER129, we characterized the spatial association of both proteins in the healthy mouse brain. Results showed that Ywhag was expressed in the mitral cell layer (Fig. 7a) and ubiquitously expressed throughout the brain (Fig. 7b). Brain regions with high PSER129 content often had high Ywhag content (Fig. 7b, Yellow color shows overlap), but the overlap was not complete and in several brain sections, particularly the cerebellum, the overlap was rare. Brain regions previously found to have high PSER129 content (Fig. 1a, c) were found to have overlapping Ywhag, with high overlap in the dentate gyrus, AOB, and substantia nigra reticulata (Fig. 7c).

In the OB, mitral cells and their apical dendrites were intensely labeled for Ywhag, while other OB layers showed moderate staining (Fig. 7d). Colocalization analysis of PSER129 positive mitral cells revealed that PSER129-Ywhag association was variable from cell-to-cell and between cellular compartments (Fig. 7d). Within the mitral cell apical dendrites, colocalization was consistently observed. However, in the soma and nucleus, the PSER129-Ywhag interaction was variable between cells, with mitral cells in two mice showing a negative correlation in these compartments (Fig. 7e).

### PSER129 positive mitral cells are c-Fos negative

**Fig. 8:**
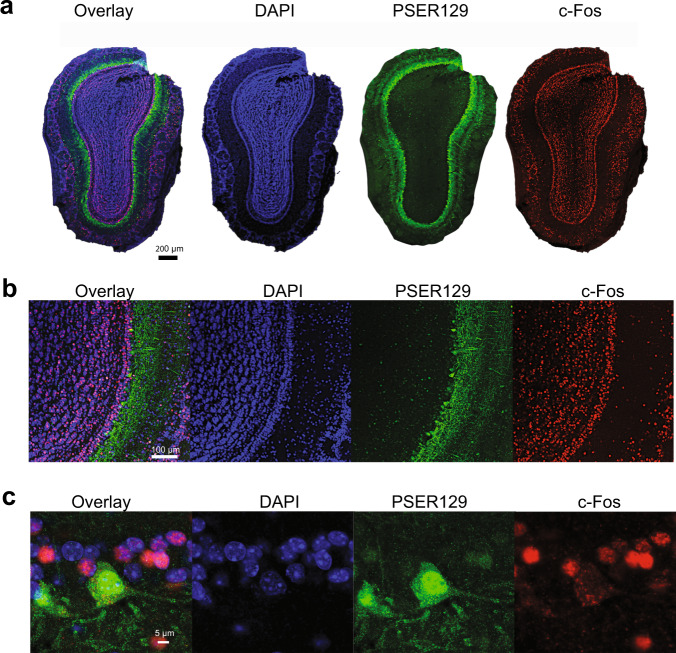
PSER129 positive mitral cells are c-Fos negative. The OB from WT mice were labeled for PSER129 and neuronal activity marker c-Fos. **a** Whole section of confocal scans of labeled OB. **b** High-magnification confocal images of the mitral cell layer. **c** Expanded high-magnification images of a single PSER129 positive mitral cell. (WT mice, *n* = 4).

The endogenous function of PSER129 remains unknown, but alpha-synuclein has been consistently implicated in playing a role in cellular vesicle processes^24,27^. Several lines of evidence suggest that neuronal activity might drive alpha-synuclein accumulation and potentially phosphorylation^28–31^. Indeed alpha-synuclein may have SNARE functions, and neuronal activity depolarization could drive accumulation/phosphorylation as SNARE becomes more active. To test this hypothesis, we used multiplex TSA to label PSER129 and the immediate early gene product c-Fos in the OB of WT mice. Nuclear c-Fos expression increases with periods of intense neuronal depolarization/activity and therefore has been used as a marker of recent neuronal activity. Results showed widespread nuclear c-Fos expression throughout the layers of the OB, including the mitral cell layer (Fig. 8a, b). High-magnification images of the mitral cell layer (Fig. 8c) revealed that PSER129 positive mitral cells lacked nuclear c-Fos expression. Cytosolic c-Fos was observed in PSER129 positive mitral cells, but the biological significance of cytosolic c-Fos is unclear. In all the animals assessed (*n* = 4), we were unable to locate a single clear instance of a PSER129-positive mitral cell expressing nuclear c-Fos.

## Discussion

The OB has been hypothesized to be one potential starting point for alpha-synuclein pathology and, thus the synucleinopathy disease process^1,11^. This contention is supported by several observations, including (1) mitral cell loss in PD OB^2,5,18,32^, (2) high incidence of LP in synucleinopathy OB^3,5,33–35^, and (3) incidental LP in OB^3^. Our inadvertent observation of PSER129 (which is usually associated with disease) in healthy mitral cells seems to support this hypothesis. However, it seems unlikely that healthy mammalian mitral cells harbor disease-causing aggregates, but instead, the phosphorylation of alpha-synuclein may have a yet undefined biological role in mitral cells. BAR-PSER129 labeling identified presynaptic vesicle machinery, fatty acid oxidation, mitochondrial oxidative phosphorylation, glycolysis, and mRNA binding and splicing as being associated with PSER129 in the OB, which is largely consistent with what is known about the function and interactome of PSER129^24,26,36^. Perturbations of these functionalities might contribute to the intracellular accumulation of PSER129. Alternatively, aggregates of misfolded alpha-synuclein might inhibit one of these functionalities resulting in accumulation and hyperphosphorylation of alpha-synuclein. Recently evidence of this was observed following alpha-synuclein pathology induction in the OB of non-human primates, which subsequently suppressed glycolytic functions^37^, suggesting that aggregates are suppressing glycolysis which we found to be functionally associated with PSER129. Factors, such as normal aging, that converge on cellular pathways identified here are likely to have particular significance for mitral cell alpha-synuclein pathology and olfactory function^5,38^.

PSER129 was closely, but not precisely, localized with PK-resistant alpha-synuclein in the mouse OB. PK resistance for alpha-synuclein occurs within pathological aggregates and via alpha-synuclein-lipid interactions^39^. Because we found evidence that PSER129 was associated with cellular vesicle machinery (Fig. 6d) and apparently monomeric on SDS-PAGE (Fig. 1e), the observed PK-resistant alpha-synuclein is likely due to lipid-alpha-synuclein interactions and not aggregation. In agreement with this interpretation, PSER129 was also not associated with ubiquitin in mitral cells, and therefore, PK-resistant alpha-synuclein observed are likely not toxic accumulations or oligomers that mitral cells are unable to clear. Interestingly, if alpha-synuclein PK resistance in mitral cells is driven by lipid interactions, then PSER129 is only proximally associated (i.e., lipid-free), suggesting that phosphorylation is a cellular signaling event that occurs prior to or following lipid-alpha-synuclein interactions, opening the possibility that PSER129 is a “molecular switch” for lipid/vesicle interactions^40^. Interestingly, we found evidence that PSER129 positive mitral cells were inactive (i.e., c-Fos negative), which does not support the hypothesis that neuronal activity is driving PSER129 and PK resistance (i.e., lipid/vesicle interactions)^28–31^.

Our data is consistent with a scenario which has previously been suggested where LP begins to form in the OB and then propagates to other brain regions in a prion-like fashion^1,11^. Mitral and tufted cells have an apical dendrite that extends to a single glomerulus, but they also extend several axons into telencephalon regions, including the amygdala^41^. These axons extend out through the olfactory tract. We observed occasional LP in the mitral cell layer of DLB and PD OB, but consistently saw dense Lewy neurites throughout the anterior olfactory nuclei of the main bulb and peduncle. In WT mice, brain regions known to be susceptible to LP in human disease frequently displayed PSER129 immunoreactivity. Notably, the PSER129 distribution that we observed here largely mirrored the known high abundance of alpha-synuclein in glutamatergic presynaptic nerve terminals in the healthy mouse brain^42,43^. If PSER129-containing brain regions are normally susceptible to pathology formation^44^, then our observations corroborate a hypothesis that olfactory cortex projections are the primary pathogenic origin of synucleinopathies^32,33,35,45–47^, and perhaps insults like excitotoxicity in the OB^48,49^ initiate the pathological processes. Similarly, if misfolding/seeding probabilities are a function of local alpha-synuclein concentration^50^, then glutamatergic processes are a high-probability site for disease initiation. Aggravating factors like infection (viral or bacterial) via nasal cavity^1^ might promote alpha-synuclein accumulation^51^ and preferentially initiate the pathological process in susceptible mitral cells.

Recent reports looking at LP distribution in PD described cases where OB LP was not present^52–54^. Two PD subtypes might exist where pathology begins either in the peripheral nervous system “body-first” or in the central nervous system “brain-first”, with LP being brainstem prominent or OB prominent, respectively^52–54^. We observed apparent LP (PK-resistant PSER129 reactivity) in all human PD OBs tested here; however, the sample size was small. Therefore, we cannot make definitive conclusions about LP distribution in the synucleinopathy OB. We can conclude that LP occurs in the mitral cell layer as observed by others^55^, and in the non-synucleinopathy OB, PSER129 reactivity can be detected in mitral cells. Future studies should assess larger human sample cohorts to provide an in-depth characterization of LP in the OB.

We initiated the present study because we had observed regionally specific anomalous PSER129 signals in healthy WT mice. Interestingly, others have previously observed this mitral cell-staining phenomenon but did not emphasize its potential importance to our understanding of synucleinopathies^56^ and the mitral cell PSER129 signal has been often dismissed as “background.” We demonstrated that the anomalous PSER129 signal was sensitive to PK digestion. In mitral cells, both diffuse and punctate structures were intensely labeled in the nucleus, cytoplasm, and often observed throughout their apical dendrite. The apparent pattern of PSER129 staining was seen in mice, rats, non-human primates, and neurologically intact humans. This suggests that the role of PSER129 in OB mitral cells is conserved across mammalian species. However, mitral cell PSER129 reactivity was not universal, as we did not observe any PK-sensitive PSER129 staining in 1 mouse and 3 healthy humans, so it’s possible that either PSER129 is generated in mitral cells driven by fluctuating cellular process or the PSER129 epitope is not accessible/preserved in some tissues. We explored many epitope retrieval methods and found that heat-mediated epitope retrieval enhanced reactivity but did not unmask reactivity in samples that showed no PSER129 reactivity to begin with, suggesting that hidden PSER129 epitopes are not responsible. In mice, we observed an “all-or-none” phenomenon where samples with little/no OB staining also had little/no staining in other brain regions. The exact reason for this phenomenon remains unclear. However, we found PSER129 interactions were strongly enriched for presynaptic glutamatergic vesicle processes and posit that fluctuations in these processes or related calcium influx^57^ could be driving the observed PSER129 in mitral cells. Alternatively, a recent report found that tandem PTM’s of alpha-synuclein PSER129 prevent detection by EP1536Y^14^. Thus, further modification of PSER129 might account for the observed loss of PSER129 signal in some brains. Postmortem dephosphorylation of PSER129 occurs rapidly in brain tissue lysates^15^, which might account for the lack of endogenous PSER129 in non-synucleinopathy postmortem human brain samples. Indeed, the only non-synucleinopathy human OB sample to show an endogenous PSER129 signal had the shortest known PMI of specimens tested here (Sample #30, PMI = 3.08 h)

In agreement with previous reports, results from our studies show that improper dilution of EP1536Y results in abundant off-target binding as determined by IHC and blotting techniques (Supplementary Fig. 2B). In contrast to previous reports, we demonstrated that endogenous PSER129 is detectably provided EP1536Y is highly dilute, and the secondary detection system is sufficiently sensitive (in this case TSA). In our hands, we found that much lower concentrations of EP1536Y than commonly used (Abcam) are necessary to ensure on-target binding and commonly employed dilutions have a high probability of generating false positive detection. Our experience showed that EP1536Y could be diluted to ~3 pg/mL (1:1,000,000 dilution) and still show on-target staining. This gives a large window of effective concentrations for this antibody, as we also observed that non-specific binding is eliminated somewhere between 1:10 K and 1:20 K dilutions (Supplementary Fig. 2B).

Synucleinopathy has not been observed as a function of aging in mammals besides humans for unknown reasons. By contrast, lesions resembling human tau tangles and amyloid plaques have been observed in mammals other than humans^58^. In the mouse OB, we found disease-associated phosphorylated tau was restricted to fibers in the glomerular, inner plexiform, and outer nerve layers, but was not observed in mitral cells (Supplementary Fig. 9). The human OB is unique amongst mammals in several aspects, including limitations in post-natal neurogenesis^58^. Human samples had the least prominent PSER129 staining of all species tested (Fig. 3a). One possibility is that PSER129 represents a protective mechanism that prevents pathological aggregation^59^ and perhaps human mitral cells are particularly deficient in this neuroprotective phosphorylation event as they age.

Studies herein are based on antibody affinity, and although we have included multiple controls (i.e., alpha-synuclein knockout mice, antibody preabsorption, primary antibody negative conditions, and WB with phosphatase treatment), we cannot completely rule out the possibility of immune mimicry^17^. However, under several IHC protocols tested (e.g., HIAR and PK), we did not observe any PSER129 immunoreactivity in any brain structure of seven alpha-synuclein knockout mice. In contrast, nearly all (14/15) of the WT mice tested had readily apparent immunoreactivity in the OB mitral layer. Using WB, we found that EP1536Y reacts to a single 15-kDa species that is abolished by phosphatase pretreatment and not observed in alpha-synuclein KOs, consistent with the detection of PSER129 (Fig. 1e, f). Furthermore, BAR-PSER129 capture samples were enriched for alpha-synuclein, as confirmed by both immunoblotting and LC-MS/MS identification (Fig. 6). Together, these observations make immune mimicry very unlikely. A second limitation of the current study is the relatively small number of human OB assessed here.

PSER129 accumulates in OB mitral cells of healthy mammals and interacts predominantly with presynaptic vesicle components. Determining the molecular and cellular factors driving the transition from endogenous PSER129 to the accumulation of abnormal PSER129 in the OB mitral cells will provide important insights into the origins of synucleinopathies.

## Methods

### Biological specimens

All rodent and non-human primate tissues were derived from studies conducted in accordance with institutional IACUC-approved protocols. M83 (B6;C3-Tg(Prnp-SNCA*A53T)83Vle/J, Jackson Labs, 6-month-old homozygous), alpha-synuclein knockout (C57BL/6N-Sncatm1Mjff/J, Jackson Labs), WT C57BL/6 mice, and Sprague-Dawley rats (SD, age 16-month-old). All animals were anesthetized (ketamine/xylazine for rodents, and ketamine/xylazine/hydromorphone for non-human primates) and transcardially perfused with PBS pH 7.4 followed by 4% paraformaldehyde (PFA) in PBS. Collected brain tissues were post-fixed in 4% PFA in PBS overnight at 4 °C, dehydrated in successive sucrose solutions in PBS (i.e.,10, 20, and 30% sucrose, w/v), and cut into 40-micron coronal sections on a freezing stage microtome. Human OB from individuals clinically diagnosed with PD were supplied by the Rush Movement Disorders Brain Bank. OB specimens from individuals without synucleinopathy (HC) were provided by Rush Alzheimer’s Brain Bank and BH Banner Health Research. Human and non-human primate OBs were sectioned following embedding in optimal cutting temperature (OCT) compound (Thermo Fisher). One human non-synucleinopathy case was obtained from Banner Health embedded in paraffin wax (Specimen details see Table 1 and Supplemental Data 6). Paraffin-embedded sections were heated in an oven to 60 °C, cleared with xylenes, and rehydrated with successive ethanol solutions from 100 to 50% prior to IHC. Once hydrated, sections were processed according to immunostaining protocols.

### Immunohistochemistry

Free-floating PFA fixed mouse sections gathered at intervals of 940 microns across the neuroaxis (i.e., OB to brainstem) were first incubated in peroxidase quenching solution (0.3% hydrogen peroxide, 0.1% sodium azide) in TBST (50 mM Tris-HCl pH 7.6, 150 mM NaCl, and 0.5% Triton X-100) for 30 min at room temperature. Sections were then rinsed twice with PBS and incubated with blocking buffer (3% normal serum, 2% BSA, TBST) for 1 h at room temperature. Sections were then incubated with either PSER129 antibody (Abcam, “EP1536Y”) diluted 1:50,000, TBX21 antibody (Santa Cruz Biotechnology) diluted 1:10,000, c-fos antibody (Abcam) diluted 1:10,000, Ywhag antibody (Abcam) diluted 1:5,000, alpha-synuclein antibody (Abcam, “EPR20535”) diluted 1:10,000, ubiquitin antibody (Abcam) 1:10,000 diluted in blocking buffer overnight at 4 °C. The next day, sections were washed twice with TBST and incubated with biotinylated anti-rabbit or anti-mouse antibodies (Vector Laboratories) diluted 1:200 in blocking buffer for 1 h at room temperature. Sections were then washed 3 times for ten minutes each in TBST and then incubated with prepared elite avidin-biotin complex (ABC) reagent (Vector Laboratories) for 1 h at room temperature. Sections were again washed two times for 10 min in TBST, and then washed two times for 10 min in sodium borate buffer (0.05 M Sodium Borate, pH 8.5). Sections were then incubated for 30 min with 1 µg/mL biotinyl tyramide (Sigma-Aldrich) and 0.003% hydrogen peroxide diluted in sodium borate buffer. Then sections were washed three times for 10 min in TBST and incubated again in the previously prepared elite ABC reagent for 1 h at room temperature. Next, sections were washed three times for 10 min in TBST. Sections were then developed using a standard nickel-enhanced 3,3′-diaminobenzidine (DAB)-imidazole protocol. Mounted sections were counterstained with methyl green, dehydrated with ethanol, cleared with xylenes, and coverslipped with Cytoseal 60 (Fisher Scientific).

For some experiments, sections were digested with PK (Thermo Fisher Scientific) prior to IHC. These sections were first mounted onto gelatin-coated slides, dried, and baked overnight at 55 °C. Slides were then placed into PBS for 10 min and then PK digestion buffer (TBS, 0.1% triton X-100, 20 µg PK / mL, warmed to 37 °C) for 20 min. Sections were rinsed with PBS, incubated with 4% PFA for 10 min, and rinsed off twice with TBST. Digested sections were then processed as stated above. For some experiments, heat-induced antigen retrieval (HIAR) was performed prior to staining. To perform HIAR, citrate buffer (10 mM sodium citrate, 0.05% Tween-20, pH 6.0) was heated in a water bath to 90 °C; slides were then immersed in this solution for 30 min and then allowed to cool to room temperature.

### TSA multiplex labeling

IHC was performed as described above with the exception that CF-488, CF-568, or CF-647 tyramide conjugates (Biotium) were substituted for biotinyl tyramide at a final concentration of 1 µg/mL. Multiplex labeling was performed by heating samples at 90 °C for 30 min in citrate buffer to remove the antibody complex following the tyramide reaction similar to what has been previously described in refs. ^60,61^. TSA multiplex labeling of PSER129 and PK-resistant alpha-synuclein was achieved by first labeling PSER129 with CF-568-tyramide. Samples were then heated in citrate buffer as described above, then allowed to cool. Samples were then washed with TBST and incubated for 3 min at 37 °C with PK digestion buffer with or without 20 µg PK/mL. Samples were next placed in 4% PFA for 10 min and then washed with TBST. Fluorescently labeled sections were mounted onto gelatin-coated slides, dried for 20 min, and covered with #1.5 glass coverslip using FluoroShield mounting medium (Sigma-Aldrich).

### BAR-PSER129

Free-floating PFA fixed mouse OB sections gathered at intervals of 120 microns were processed as described above and previously^23^. Each sample consisted of ~15–20 OB tissue sections. Sections from WT and alpha-synuclein KO mice were used. Capture conditions were conducted both with primary antibody (i.e., EP1536Y) and without primary antibody. Following the biotinyl tyramide reaction, sections were washed overnight in PBS, and then heated to 98 °C in 1 mL of crosslink reversal buffer (500 mM Tris-HCl pH 8.0, 5% SDS, 150 mM NaCl, and 2 mM EDTA) for 30 min. Samples were then vortexed vigorously, heated for an additional 15 min, and centrifuged for 30 min at 22,000 × *g*. The supernatant was diluted 1:10 in dilution buffer (50 mM Tris-HCl pH 8.0, 1% triton X-100, 150 mM NaCl) and incubated with 25 µg of streptavidin magnetic beads (Thermo Fisher Scientific) overnight at 4 °C with nutation. The following day, beads were collected on a magnetic stand, washed three times for 1 h each with wash buffer (50 mM Tris-HCl pH 8.0, 1% triton X-100, 0.1% SDS, and 150 mM NaCl), and then one time with TBST for 1 h. Beads were then collected and boiled in 40 µl sodium dodecyl sulfate-polyacrylamide gel electrophoresis (SDS-PAGE) sample buffer containing 5% beta-mercaptoethanol for 10 min and mixed vigorously. About 38 µl were run a distance of ~1 cm into an SDS-PAGE gel (Fisher Scientific); the gel was fixed (1 h in 50% ethanol and 10% acetic acid), stained with Coomassie blue, and the entire lane excised for subsequent trypsin digestion and LC-MS/MS.

### LC-MS/MS

Gel bands were washed in 100 mM ammonium bicarbonate (AmBic)/acetonitrile (ACN) and reduced with 10 mM dithiothreitol at 50 °C for 45 min. Cysteines were alkylated with 100 mM iodoacetamide in the dark for 45 min at room temperature (RT). Gel bands were washed in 100 mM AmBic/ACN prior to adding 1 µg trypsin (Promega #V5111) for overnight incubation at 37 °C. Supernatant-containing peptides were collected into a new tube. Gel pieces were washed with gentle shaking in 50% ACN/1% FA at RT for ten minutes, and the supernatant was collected in the previous tubes. The final peptide extraction step was done with 80% ACN/1% FA and 100% ACN, and all supernatant was collected. The peptides were dried in a SpeedVac and reconstituted with 5% ACN/0.1% FA in water before injecting them into the LC-MS/MS.

Peptides were analyzed by LC-MS/MS using a Dionex UltiMate 3000 Rapid Separation nanoLC coupled to an Orbitrap Elite Mass Spectrometer (Thermo Fisher Scientific Inc.). Samples were loaded onto the trap column, which was 150 μm × 3 cm in-house and packed with 3 µm ReproSil-Pur® beads. The analytical column was a 75 µm × 10.5 cm PicoChip column packed with 3 µm ReproSil-Pur® beads (New Objective, Inc. Woburn, MA). The flow rate was kept at 300 nL/min. All fractions were eluted from the analytical column at a flow rate of 300 nL/min using an initial gradient elution of 5% B from 0 to 5 min, transitioned to 40% over 100 min, 60% for 4 min, ramping up to 90% B for 3 min, holding 90% B for 3 min, followed by re-equilibration of 5% B at 10 min with a total run time of 120 min. Mass spectra (MS) and tandem mass spectra (MS/MS) were recorded in positive-ion and high-sensitivity mode with a resolution of ~60,000 full-width half-maximum. The 15 most abundant precursor ions in each MS1 scan were selected for fragmentation by collision-induced dissociation (CID) at 35% normalized collision energy in the ion trap. Previously selected ions were dynamically excluded from re-selection for 60 s. The mass range for MS/MS was 475–2000 m/z with 60,000 and 30,000 resolutions for MS1 and MS2, respectively. The collected raw files spectra were stored in .raw format.

Proteins were identified from the MS raw files using the Mascot search engine (Matrix Science, London, UK. version 2.5.1). MS/MS spectra were searched against the SwissProt mouse database. All searches included carbamidomethyl cysteine as a fixed modification and oxidized methionine, deamidated asparagine and aspartic acid, and acetylated N-terminal as variable modifications. Biotin (K) and Biotin(N-term) were set as variable modifications for biotinylation detection. Three missed tryptic cleavages were allowed. A 1% false discovery rate cutoff was applied at the peptide level. Only proteins with a minimum of one peptide above the cutoff were considered for further study. Identified peptides/proteins were visualized by Scaffold software (version 5.0, Proteome Software Inc., Portland, OR).

### Pathway analysis

Full STRING network analysis was conducted on the 125 PSER129-interacting proteins identified by BAR. For STRING, protein identifiers' minimum required interaction score was set to 0.7 (i.e., high confidence) and singlet nodes (i.e., no interactions) were discarded by selecting “hide disconnected nodes in the network”. Data for all functional and physical protein associations were used. The resulting STRING network was exported to Cytoscape v.3.9.1 for Markov Cluster Algorithm (MCL) clustering and pathway analysis. Consensus terms for each cluster was determined by gene ontology (GO) most enriched pathways (i.e., lowest FDR). The final annotated interaction map was manually compared with alpha-synuclein interactions previously identified in vivo using proximity labeling methods^24^.

### Spot blotting

Prior to MS, 1 µl of streptavidin bead eluent was spotted onto a methanol-activated polyvinylidene difluoride (PVDF) membrane and allowed to dry completely. The membrane was then reactivated in methanol, washed in water, and post-fixed in 4% PFA for 30 min. Blots were then rinsed with TBST (20 mM Tris-HCl pH 7.6, 150 mM NaCl, 0.1% Tween-20) and placed in blocking buffer (TBST and 5% BSA) for 1 h at room temperature. To detect biotinylated proteins, blots were incubated with ABC (VectorLabs) diluted 1:10 in a blocking buffer for 1 h at room temperature. To detect alpha-synuclein, blots were incubated overnight with SYN1 diluted 1:2000 in blocking buffer, washed, and then incubated with anti-mouse HRP conjugate diluted 1:6000 in blocking buffer (Cell Signaling). All membranes were washed in high stringency wash buffer (20 mM Tris-HCl pH 7.6, 400 mM NaCl, 0.1% Tween-20) and imaged using enhanced chemiluminescence (ECL) substrate (Bio-Rad, product # 1705060) and Chemidoc imager (Bio-Rad).

### Western blotting

Proteins were extracted from unlabeled fixed floating tissue sections as described above. Extracted proteins were precipitated by chloroform-methanol^62^ and re-dissolved in 5% SDS. Protein concentrations were determined using bicinchoninic acid assay (BCA, Thermo Fisher) and 20 µg of total protein was separated on 4–12% Tris-glycine gel. Proteins were blotted onto PVDF membranes, membranes were then post-fixed with 4% PFA for 30 min, dried, and reactivated in methanol for immunoblotting. Membranes were incubated for 1 h in blocking buffer (TBST with 5% non-fat milk and 0.5% polyvinylpyrrolidone) and then with either SYN1 (diluted 1:2000) or PSER129 (diluted 1:20,000) antibodies diluted in blocking buffer overnight at 4 °C. The following day, membranes were washed 3 × 10 min in TBST and incubated with anti-rabbit (Invitrogen, 1:20,000) or anti-mouse (Cell Signaling, 1:6000) HRP conjugates for 1 h at room temperature. Membranes were then washed for 3 × 10 min in TBST. Membranes were imaged using an ECL substrate and chemiluminescence imager (Bio-Rad). Five microliters of a broad range molecular weight standard (Bio-Rad, product # 1610394) was used to determine the approximate molecular weight of separated proteins.

### Calf intestine alkaline phosphatase (CIAP) treatment

Proteins were separated and blotted as described above. All samples to be tested were loaded in duplicate on the same gel and blotted to the same membrane. Following PFA fixation, membranes were stained with ponceau S and cut to separate the duplicates. Both membranes were blocked as previously described, and then washed for 3 × 5 min with TBST. One blot was incubated at 37 °C for 2 h with 10 mL CIAP buffer (100 mM NaCl, 10 mM MgCl2, and 50 mM Tris-HCl pH 7.9) containing 1000 units CIAP enzyme (Promega), and the other blot was incubated with CIAP buffer without CIAP enzyme. Blots were then washed for 3 × 5 min with TBST and immunoprobed as described above. Blots were imaged in tandem.

### Microscopy

All samples were imaged on an inverted confocal microscope (Nikon A1R). Whole slide bright-field images using a 4X or 10X objective were acquired for DAB-labeled sections. Confocal images were acquired for fluorescently labeled sections using a 60X oil immersion objective. Deconvolution of confocal images and colocalization analysis was performed using Nikon elements software. Colocalization data were plotted using Prism (GraphPad Software).

## Supplementary information

Supplementary Information

NPJ Report summary

Supplemental Data 6

Supplementary Data 4

Supplementary Data 5

Supplementary Data 1

Supplementary Data 2

Supplementary Data 3

## Data Availability

LC-MS/MS data have been deposited to the ProteomeXchange Consortium via the PRIDE partner repository with the dataset identifier PXD040392^63^.
