# Supplementary material for: Distribution of phosphorylated alpha-synuclein in non-diseased brain implicates olfactory bulb mitral cells in synucleinopathy pathogenesis
Source: NPJ Parkinsons Dis. 2023 Mar 25;9:43. doi: 10.1038/s41531-023-00491-3 (PMC10039879; doi:10.1038/s41531-023-00491-3)
Supplement: Supplementary file 1 — Supplementary Information [file 41531_2023_491_MOESM1_ESM.docx]

**Supplementary Information for**

Distribution of phosphorylated alpha-synuclein in non-diseased brain implicates olfactory bulb mitral cells in synucleinopathy pathogenesis

Bryan A. Killinger^a^, Gabriela Mercado^b^, Solji Choi^a^, Yaping Chu^C^, Tyler Tittle^a^, Patrik Brundin^b,d^, and Jeffrey H. Kordower^C^

^a^Graduate College, Rush University Medical Center, Chicago Illinois 60612

^b^ Parkinson’s disease Center, Department of Neurodegenerative Science, Van Andel Institute, Grand Rapids MI 49503

^c^ ASU-Banner Neurodegenerative Disease Research Center (NDRC), Arizona State University, Tempe Arizona 85287

^d^Pharma Research and Early Development (pRED), F Hoffman-La Roche, New York, USA

*Bryan A. Killinger

**Email:**  [bryan_killinger@rush.edu](mailto:bryan_killinger@rush.edu)

**This PDF file includes:**

Supplementary Text

Supplementary Figures 1 to 9

Legends for Supplementary Data 1 to 6

Supplementary Table 1

**Other supplementary materials for this manuscript include the following:**

Datasets 1 to 6

**
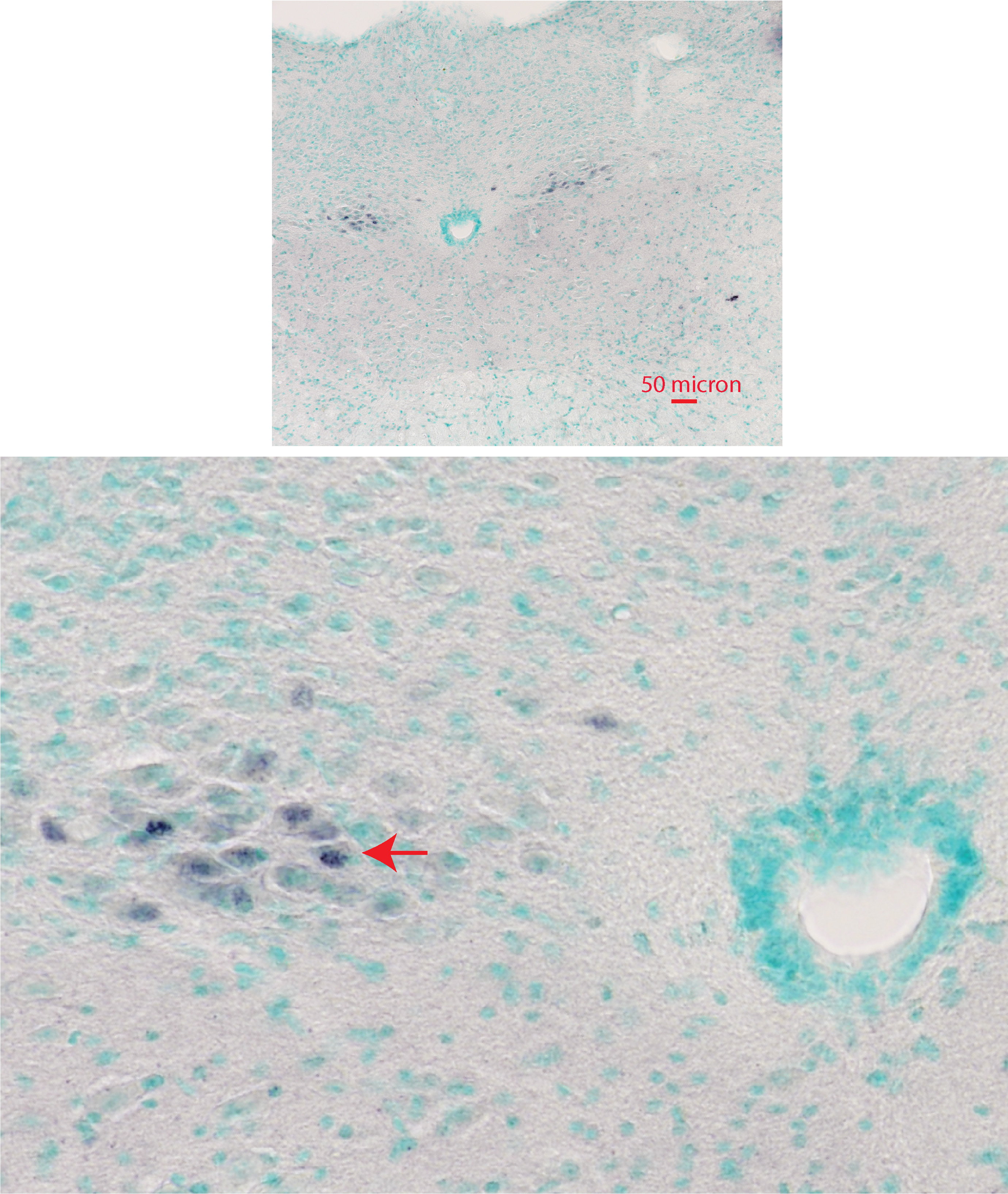
**

**Supplementary Figure 1.** PSER129 immunoreactivity in the dorsal motor nucleus of the vagus (DMNV). PSER129 staining was absent from the brainstem and cerebellum of wildtype mice except for immunoreactivity of a small group of cells in the DMNV shown here. Red arrow highlights the reactive cells.


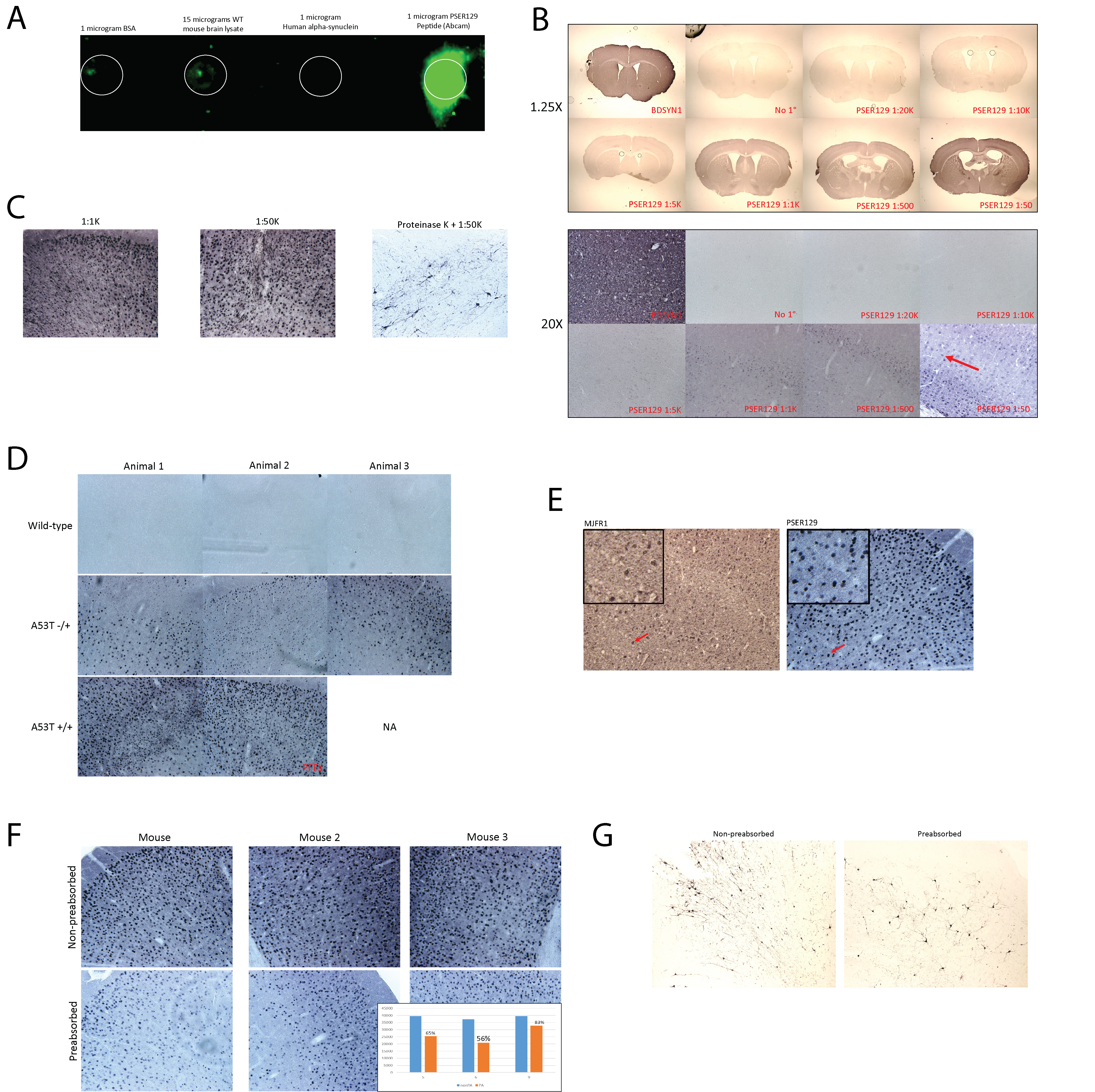


**Supplementary Figure 2**. Validation Experiments in M83 mice and WT mice. (A) Dot-blot demonstrates EP1536Y reactivity against several samples of interest. (B) EP1536Y dilution experiments. Brain sections from wild-type mice were probed with the antibody BDSYN1 (total synuclein) or several dilutions of EP1536Y and developed with DAB or nickel enhanced DAB. Low and high magnification images of the resulting reactivity. Higher dilutions were required to avoid perfuse reactivity in the brain. (D) PSER129 reactivity in WT and A53T alpha-synuclein overexpressing mice (M83). At high dilutions, PSER129 reactivity was seen throughout the M83 brain. (E) Antibody MJFR1 only reacts to human alpha-synuclein. M83 mice show similar tissue distribution of human alpha-synuclein as PSER129 tissues distribution. This suggests that expressed human alpha-synuclein is phosphorylated. (F) Preabsorption of EP1536Y antibody. The antibody was preaborbed against excesses alpha-synuclein phosphopeptide (abcam). The preabsorbed antibody was then used to stain brain sections from M83 mice not bearing alpha-synuclein pathology. Results show a reduction in signal following preabsorbtion, but not a loss of staining, typical of monoclonal antibody reactivity. (G) Preabsorbed antibody was used to stain proteinase K treated M83 mouse brains baring alpha-synuclein pathology. Similarly, pathology detection was inhibited with preabsorbtion, but not eliminated.


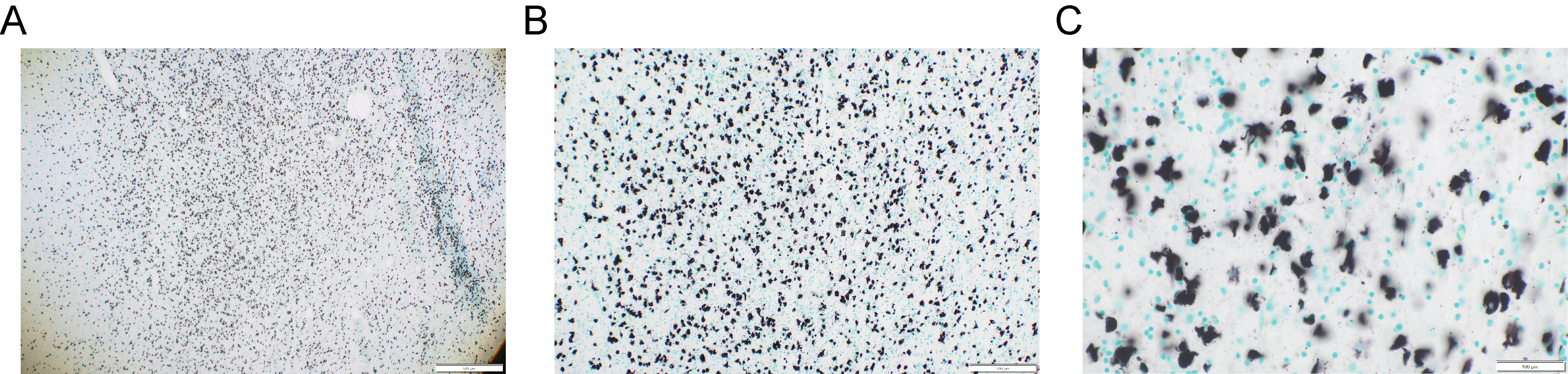


**Supplementary Figure 3.** PSER129 content in multiple systems atrophy(MSA) tissue section used for positive control in Fig. 1. Free-floating striatal section from MSA brain stained for PSER129 using antibody EP1536Y. Sections were counterstained with methyl green. Images acquired with a (A) 4X (B) 10X and (C) 20X objective. Dense glial cytoplasmic inclusions were observed predominantly in white matter of the tissue section.


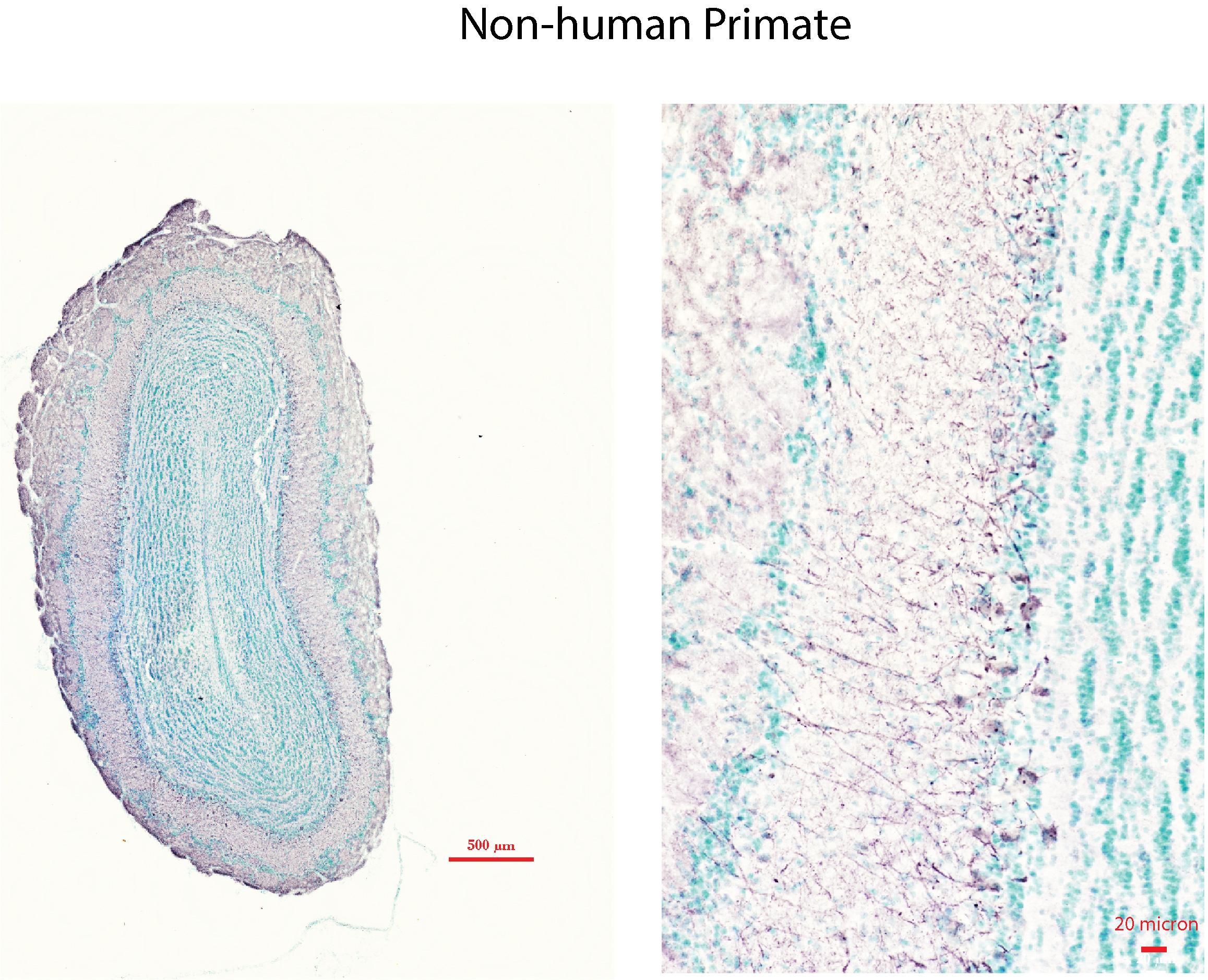


Supplementary Figure 4. Antibody pSyn#64 reactivity in OB of a healthy non-human primate. Formalin fixed floating coronal sections from a cynomolgus monkey were incubated with mouse monoclonal antibody pSyn#64 (FuJIFILM Wako Pure Chemical Corporation) diluted 1:10K and detected using tyramine signal amplification. Sections were counterstained with methyl green. The left image depicts a whole section scan, and the left image depicts an image taken with a 20X objective. Scale bars are depicted on each image.

<


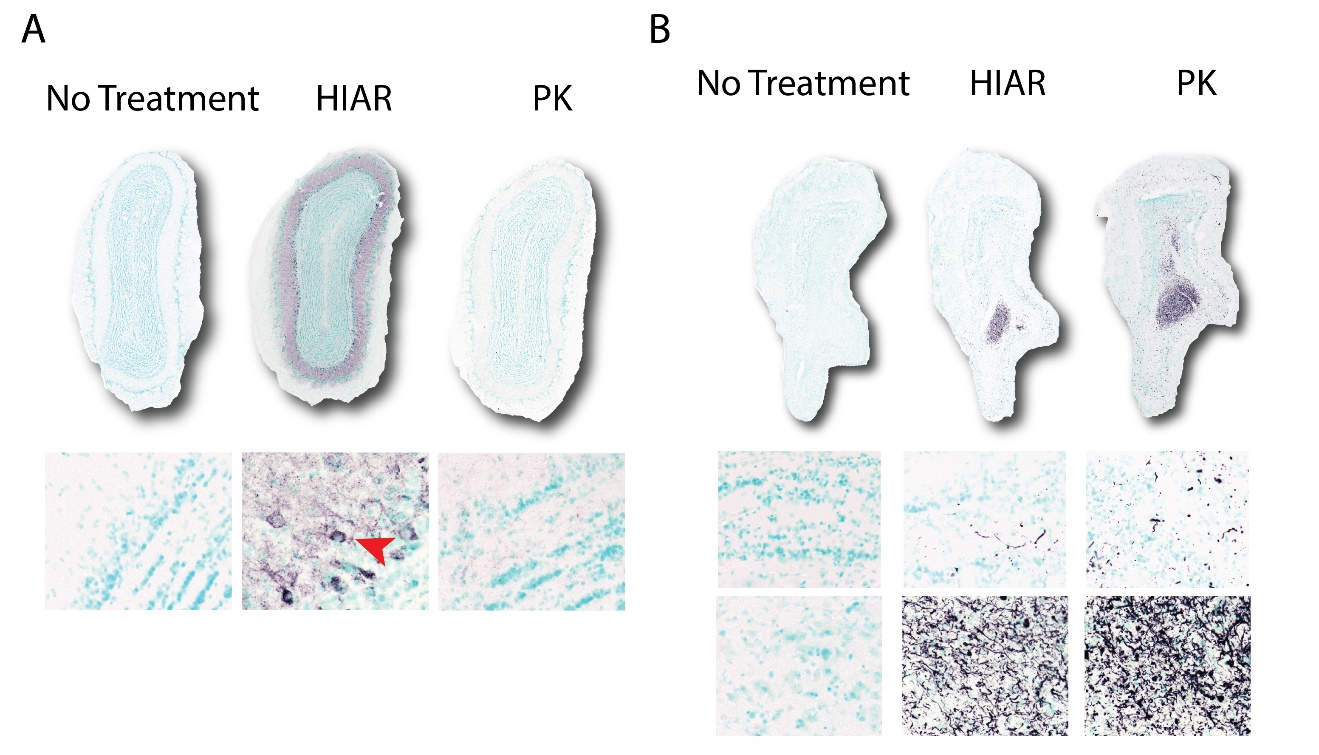


**Supplementary Figure 5.** Detection of PSER129 is dependent on antigen retrieval techniques. OB sections from a non-human primate (A) and individual with Parkinson’s disease (B) were immunostained using the EP1536Y antibody under different tissue processing conditions. For the “no treatment” condition tissues were stained as floating sections without antigen retrieval. For “HIAR” tissues were mounted onto slides and heated to ~95°C in citrate buffer for 30 min. For “PK” samples were mounted onto slides and treated with 20 µg/mL proteinase K for 10 min at 37°C. Resulting EP1536Y immunoreactivity for each condition is depicted. Red arrow highlights PSER129 reactivity in mitral cells.


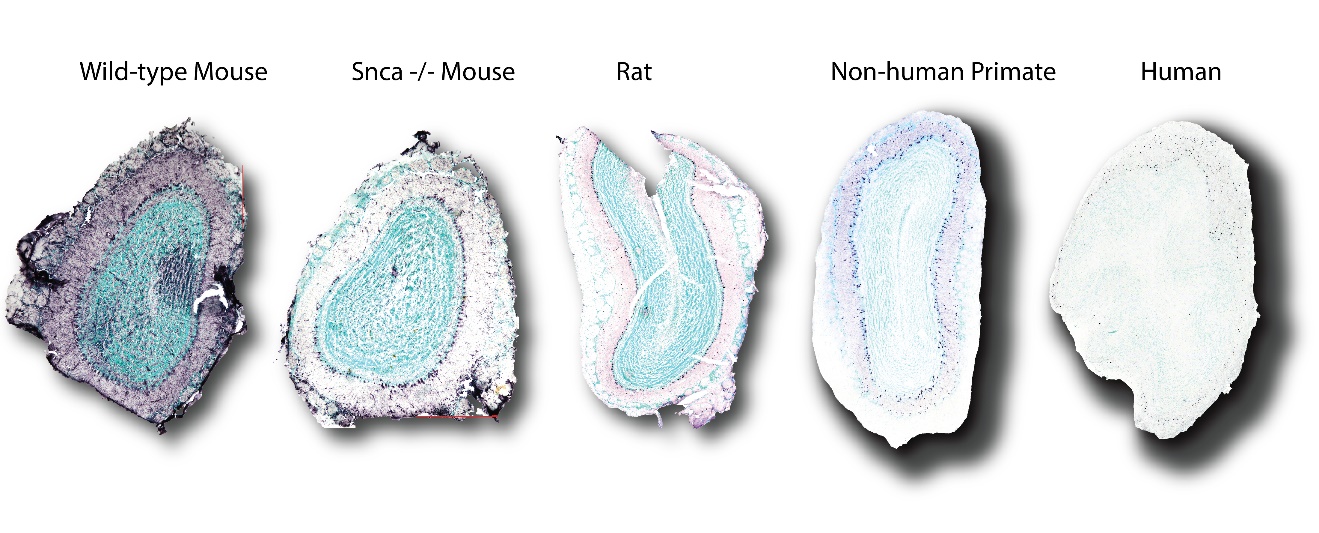


**Supplementary Figure 6.** TBX21 reactivity in the OB. Immunoreactivity of mitral cell marker TBX21 in OB specimens from each species assessed in this study. TBX21 reactivity was observed in the mitral cell layer across species. High background was observed in the mouse specimens because the anti-TBX21 antibody was of mouse origin.


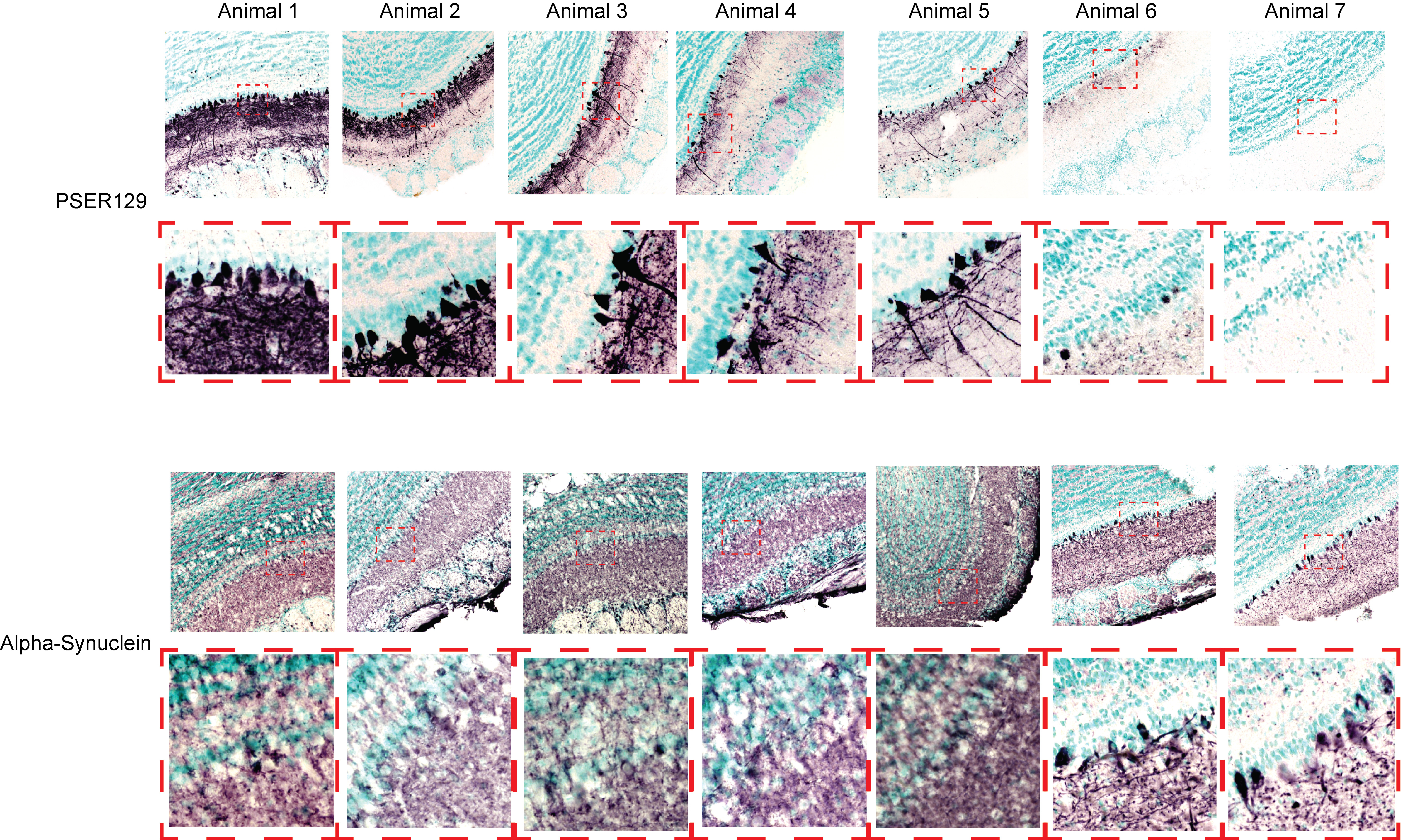


**Supplementary Figure 7.** PSER129 and Alpha-synuclein in OB mitral cell layer of WT mice. PSER129 and alpha-synuclein were stained in WT mice OB’s using antibodies EP1536Y (Abcam) and SYN1 (BDbiosciences), respectively. Nickle-DAB was used as a chromogen and sections were counterstained with methyl green. Images were acquired using a 20X objective. Enlarged images of mitral cell layer are depicted in bottom panels and position of enlarged image denoted by a red dotted box. N=7 WT mice.


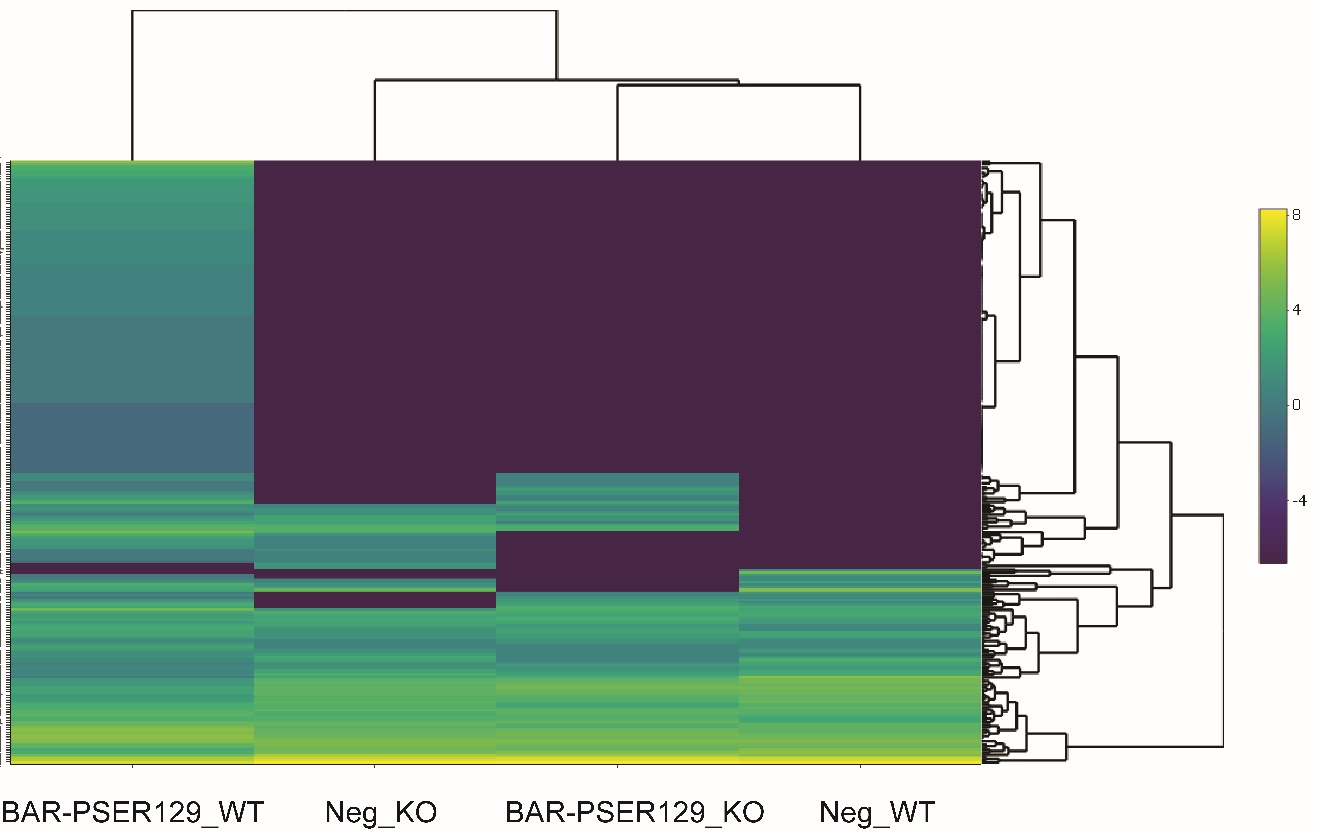


**Supplementary Figure 8.** Heatmap of proteins identified in each BAR sample. BAR was conducted on tissue sections from non-diseased wildtype (WT) and alpha-synuclein knockout (KO) mice. BAR was conducted with anti-PSER129 (BAR-PSER129) and without primary antibody (Neg). Log2 values of relative protein abundance.

**
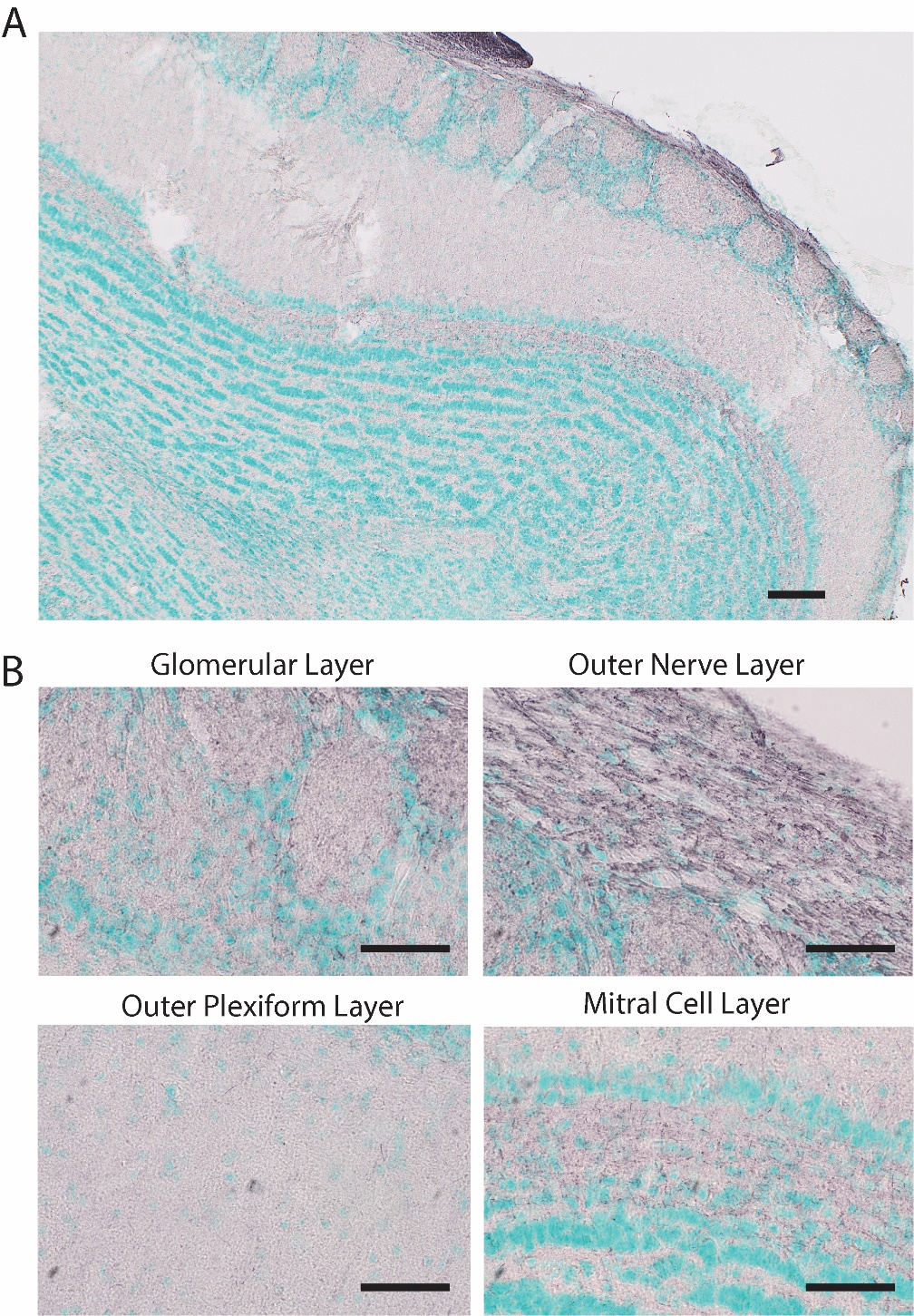
**

**Supplementary Figure 9**. Phospho-Tau immunoreactivity in the mouse olfactory bulb. 40-micron floating olfactory bulb sections were incubated with anti-phospho-tau antibody EPR2731 (Abcam) diluted 1:10K and detected using ABC with Nickle-DAB Chromogen. Sections counterstained with methylgreen. (A) Representative image taken using a 4X objective. (B) Representative images taken using a 10X objective. Depicted are the glomerular layer, outer nerve layer, outer plexiform layer, and the mitral cell layer. Prominent staining observed in the inner plexiform layer, outer nerve layer, and the glomerular layer. For (A) scale bar = 200 microns. For (B) scale bars = 100 microns.

| **Antibody** | **Source** | **Specificity** | **Immunogen** | **Epitope** | **Isotype** | **Host** | **Catalog#** | **Clonality** |
| --- | --- | --- | --- | --- | --- | --- | --- | --- |
| EP1536Y | Abcam | PSER129 | Synthetic peptide | MPSEE | IgG | Rabbit | ab51253 | Monoclonal |
| pSyn#64 | Wako Chemicals USA | PSER129 | Synthetic peptide | AYEMPSEEGYQ | IgG2b | Mouse | 014-20281 | Monoclonal |
| 4B10 | Santa Cruz Biotechnology | TBX21 | full length recombinant murine TBX21 | NA | NA | Mouse | sc-21749 | Monoclonal |
| Syn1 (clone 42) | BD Biosciences | Alpha-synuclein | Rat Alpha-Synuclein aa. 15-123 | ATGFVKKDQL | IgG1 | Mouse | 610787 | Monoclonal |
| EPR20535 | Abcam | Alpha-synuclein | Recombinant full-length protein | Unknown | IgG | Rabbit | ab212184 | Monoclonal |
| NA | Abcam | cfos | Recombinant full-length c-fos | Unknown | IgG | Rabbit | ab190289 | Polyclonal |
| NA | Abcam | YWHAG | Recombinant fragment, aa 1-179 | Unknown | IgG | Rabbit | ab155050 | Polyclonal |
| EPR2731 | Abcam | Tau, phospho S396 | Synthetic peptide | Unknown | IgG | Rabbit | ab109390 | monoclonal |

Supplementary Table 1. Primary Antibodies

**Legends for supplemental data**

Supplementary Data 1 (separate file). Scaffold protein report for BAR experiments. Protein level data exported from Scaffold. Sample key; 19 = BAR-PSER129-WT, 15 = Neg-WT ,12 = BAR-PSER129-KO , 18 = Neg-KO.

Supplementary Data 2 (separate file). Peptide report for BAR experiments. Peptide level data exported from Scaffold. Sample key; 19 = BAR-PSER129-WT, 15 = Neg-WT ,12 = BAR-PSER129-KO , 18 = Neg-KO.

**Supplementary Data 3 (separate file).** Proteins detected exclusively in BAR-PSER129-WT. 125 proteins detected only in the BAR-PSER129-WT sample. Accession Number, gene name, and normalized total spectra value.

Supplementary Data 4 (separate file). Enrichment results from STRING. The 125 proteins were input into STRING, and 783 significantly enriched pathways were determined.

Supplementary Data 5 (separate file). Enrichment results from gprofiler. The 125 proteins were ranked by relative abundance and then enrichment determined by gprofiler. 436 significantly enriched pathways were determined.

Supplementary Data 6 (separate file). Human OB specimens were provided by three brain banks. Banner Sun Health, Rush Alzheimer’s Disease Center, and Rush Movement Disorders Brain Bank. Detailed clinical and post-mortem information is included in this excel sheet.
